# A Genome-Scale Integration and Analysis of *Lactococcus lactis* Translation Data

**DOI:** 10.1371/journal.pcbi.1003240

**Published:** 2013-10-10

**Authors:** Julien Racle, Flora Picard, Laurence Girbal, Muriel Cocaign-Bousquet, Vassily Hatzimanikatis

**Affiliations:** 1 Laboratory of Computational Systems Biotechnology, Ecole Polytechnique Fédérale de Lausanne (EPFL), Lausanne, Switzerland; 2 Swiss Institute of Bioinformatics (SIB), Lausanne, Switzerland; 3 Université de Toulouse; INSA, UPS, INP; LISBP, Toulouse, France; 4 INRA, UMR792 Ingénierie des Systèmes Biologiques et des Procédés, Toulouse, France; 5 CNRS, UMR5504, Toulouse, France; Princeton University, United States of America

## Abstract

Protein synthesis is a template polymerization process composed by three main steps: initiation, elongation, and termination. During translation, ribosomes are engaged into polysomes whose size is used for the quantitative characterization of translatome. However, simultaneous transcription and translation in the bacterial cytosol complicates the analysis of translatome data. We established a procedure for robust estimation of the ribosomal density in hundreds of genes from *Lactococcus lactis* polysome size measurements. We used a mechanistic model of translation to integrate the information about the ribosomal density and for the first time we estimated the protein synthesis rate for each gene and identified the rate limiting steps. Contrary to conventional considerations, we find significant number of genes to be elongation limited. This number increases during stress conditions compared to optimal growth and proteins synthesized at maximum rate are predominantly elongation limited. Consistent with bacterial physiology, we found proteins with similar rate and control characteristics belonging to the same functional categories. Under stress conditions, we found that synthesis rate of regulatory proteins is becoming comparable to proteins favored under optimal growth. These findings suggest that the coupling of metabolic states and protein synthesis is more important than previously thought.

## Author Summary

Post-transcriptional regulation is important for the understanding of gene expression control. Our work is a genome-scale analysis of the translation steps of protein synthesis from transcripts. We have developed a mathematical model to integrate and analyze experimental ribosome density of hundreds of transcripts of *Lactococcus lactis*, providing robust estimation of polysome sizes. Using a mechanistic approach we have modeled for the first time in bacteria the protein synthesis rate for each gene and determined by control analysis the limiting rate between initiation, elongation and termination. Highly expressed proteins belonged to the group of the proteins with high synthesis rate and were controlled by elongation. Unexpectedly, a significant number of genes under elongation limitation were found although initiation was generally believed to be limiting. In addition, we showed that translation rate and control were in agreement with cellular requirements in cells growing in optimal environment but also in cells under nutritional limitation. This work provided a better understanding of translational regulation in bacteria and demonstrated how protein synthesis control was closely related to cellular metabolic states.

## Introduction

**Figure 1:**
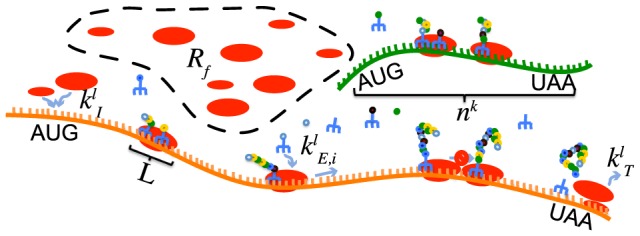
Scheme of translation process. *R_f_* is the number of free ribosomes, *k_I_ k_E_ k_T_* are rate constants of translation steps, *L* is the number of codons covered by one ribosome and *n^k^* is the number of codons of the gene coding sequence.

Translation is involved in the multi-layer process of the gene expression and allows the transfer of gene coding information from RNA to protein through ribosome action. Translation is composed of three successive steps: initiation, elongation and termination. During initiation, a ribosome binds to an mRNA at the ribosome-binding site to initiate translation at the beginning of the coding sequence (Figure 1). Next, the ribosome moves forward on the mRNA reading the sequence of codons and synthesizes the corresponding sequence of amino acids. Several ribosomes are translating simultaneously the same mRNA molecule, and this mRNA-ribosome complex is called polyribosomes or polysomes. When a ribosome reaches the stop codon translation ends with the termination step during which the native protein and the ribosome are released from the mRNA.

Using the polysome size, we can define the ribosomal density, ρ (see *Materials and Methods*). It goes from 0 (empty mRNA) to 1 (mRNA full of ribosomes) and takes into account the length of the gene, its polysome size and the size of a ribosome. The ribosomal density influences translation efficiency: it is generally postulated that the higher the number of bound ribosomes, the greater the number of protein molecules produced from a transcript. However, we observed in our modeling and computational studies that this is not in general true because it appears that ribosome traffic jam can emerge and slow down translation [1], [2].

In prokaryotes, Hatzimanikatis and co-workers investigated the relation between protein synthesis rate, rate limitation and ribosomal density [1]–[4]. They used a kinetic model for translation based on works from MacDonald and Gibbs [5] and Heinrich and Rapoport [6]. In these recent studies [1]–[4], the model was extended to account for all elementary steps of translation in *Escherichia coli* and the authors applied a metabolic control analysis framework to determine when translation is initiation-, elongation- or termination-limited. They found that translation rate increased with increasing ribosomal density, reached a maximum and then decreased. For almost the entire range of ribosomal densities, the translation kinetics was either initiation- or elongation-limited, with the maximum protein synthesis occurring at a ribosomal density corresponding to elongation-limitation [1], [2].

However, these studies were based only on modeling and simulations, since no experimental data for genome-wide *in vivo* ribosomal densities was yet available in prokaryotes. Indeed, up to now, such data was only available in eukaryotes [7]–[10]. This nevertheless changed recently: the ribosomal density of each mRNA present in a cell was for the first time experimentally measured in a bacterium (*Lactococcus lactis*) [11]. In these studies, a great variability of ribosomal densities was observed. There, Girbal, Cocaign-Bousquet and co-workers estimated the relative contribution of various factors in explaining these polysome data (such as mRNA concentration, mRNA half-life, gene length, CAI and specific codon sequences).

The aim of this study was to analyze protein synthesis rate and control at the genome-wide scale in a prokaryotic organism, the bacterium *Lactococcus lactis*. One of the challenges was to estimate ribosomal density from genome-wide polysome size measurements due to the complexity that arises from the fact that all components are mixed in a single compartment in prokaryotes, potentially leading to simultaneous transcription and translation. Here we further integrated and analyzed these data, along with similar studies from different physiological conditions, and we designed a data analysis procedure to estimate robustly the polysome density based on the experimental data. From these values of estimated polysome densities we then used a mechanistic model to determine the protein synthesis rates for individual genes and we quantified the rate limiting steps of translation. These results were further analyzed in view of the gene functionalities.

## Materials and Methods

### Experimental data

*L. lactis* subsp. *lactis* IL1403 was grown in batch cultures in a modified chemically defined medium in exponential conditions (growth rate of 0.88 h^−1^) and isoleucine starvation conditions (growth rate of 0.05 h^−1^) [12]. Translatome experiments were performed to determine genome-wide ribosomal density and ribosome occupancy (fraction of mRNA molecules engaged in translation) in normal and stress conditions (Figure S1) [11], [13]. Briefly, after translation arrest and cell disruption, size fractionation of mRNA-ribosome complexes on sucrose gradient was processed. In the elution fractions corresponding to different polysome sizes, total RNA was extracted and hybridized to microarrays. For each microarray series, normalization steps including intra-series and inter-series normalization, correction of intensity values to the total RNA quantity and their centering reduction were performed to determine the number of bound ribosomes on each mRNA molecules. The fraction of ribosomes engaged in translation, noted 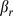, was experimentally estimated by area integration of the polysomal profile and equaled to 0.61 in normal and stress conditions [11]. Under similar conditions, genome-wide transcriptomic-based methods were previously used to determine mRNA concentrations and mRNA half-lives in *L. lactis* [11], [13].

### Model of protein synthesis

Translation was modeled by considering the individual motion of the ribosomes along the mRNA chain (Figure 1) [3], [6]. The first step is the binding of the ribosome to the initiation site. The ribosome is then considered as a hard body that covers a number *L* of codons, (considered to be 10 in our study [14]) that can move along the mRNA. At the final stage, when reaching the termination codon, the ribosome releases its newly formed protein and the ribosome unbinds from the mRNA. For the mathematical formulation of the model, we consider the mass balance equations for the codons occupied by the front of a ribosome:
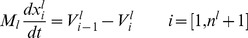
(1)where 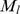 is the copy number of the mRNA species *l* engaged in translation, 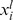 is the probability of having a ribosome front in codon *i* of mRNA species *l*, 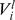 are the various fluxes of transitions of the ribosomes, defined in the following way:
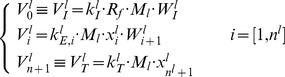
(2)The initiation, elongation and termination rate constants are given by 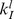, 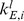 and 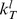 respectively. 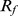 represents the free ribosomes. 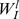 is the probability that the initiation site of mRNA species *l* is empty, and 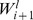 is the probability that codon 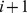 is empty knowing that the front of a ribosome is on codon *i*. These probabilities are given by:
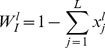
(3)

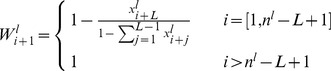
(4)It is therefore needed to solve for each species 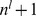 nonlinear ordinary differential equations. Note however that we are looking for the steady state solutions of the system.

The absolute protein synthesis rate of gene species *l* is given by 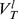 of equation 2. This corresponds to the rate of synthesis of proteins from this species from all mRNA copies engaged in translation of this species. The experiment could not determine the absolute concentrations of the mRNA species, but it was possible to obtain relative concentrations between the species. We can therefore get a “normalized absolute protein synthesis rate” for each species and compare their values (see *Normalized absolute protein synthesis rate* section below). The specific protein synthesis rate is defined as the rate of synthesis of proteins per mRNA copy. It is therefore given by:
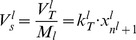
(5)The ribosomal density as defined in [1], [2], noted 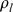, is proportional to polysome size, the number of ribosomes bound to a single mRNA molecule.
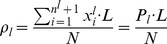
(6)

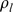
 varies therefore between zero (no ribosome loaded on mRNA) and one (full coverage of the mRNA by ribosomes). 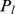 is the polysome size of mRNA species *l*.

In these equations we have kept the elongation rate constant as codon-dependent (i.e. 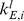 in equation 2) however for the rest of this study we will use an averaged value, codon-independent, noted simply 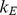. For this value, we used the cell-averaged value of 23 amino acids per second and per ribosome, which we computed for *L. lactis* (see Text S1). In order to determine the value of the free parameters 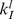 and 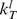 for each gene we made an assumption: the steady state protein synthesis rate of each mRNA is maximized by the cell under the constraint, gene specific, given by the polysome size of each mRNA (estimated experimentally). Indeed, protein synthesis is a very expensive process and we can therefore assume the cell has optimized this process to be the most efficient possible, reducing the cost of wasted energy (otherwise the cell would need to use more ribosomes and mRNA copies in order to reach the same production of proteins). Note that this assumption is equivalent to having the termination rate constant as big as possible, so that it is usually not rate limiting.

With this assumption and knowing the experimental polysome size of each gene [11], we could determine the unique pair of initiation and termination rate constants that was resulting in this polysome size and maximum specific protein synthesis rate, by solving the system of equations 1–4 and 6 together [15]. Briefly, this is done with the following principle: the termination rate constant is first fixed to a high, non-limiting value; then the initiation rate constant is increased, starting from 0 and the polysome size is recorded in function of the initiation rate constant; if the target polysome size is reached the wished pair of initiation/termination is obtained. However there is the possibility that the target polysome size is not reached and that any further increase in initiation rate constant does not lead to further increase in polysome size. In such a case, it is now the termination rate constant that is varied, by decreasing its value until the target polysome size is reached.

For the computations an additional assumption was made: all ribosomes on the gene were considered to be active. In eukaryotes, it has been observed that some ribosomes could bind in the 5′ UTR [16] and would therefore not really be active. However 5′ UTR are usually shorter in prokaryotes and present fewer regulations, therefore this should only have a small impact.

#### Normalized absolute protein synthesis rate

It was explained above how we can get the specific protein synthesis rate from the model, when knowing the ribosomal densities of the genes. The specific synthesis rate describes how many proteins are synthesized per second and per mRNA copy of the gene. But some genes will be present in more mRNA copies than other and it then gives useful information to also estimate the absolute protein synthesis rate, which tells how many proteins are synthesized per second in the cell, for each gene species. As seen earlier, not all mRNA copies of a gene are actively translating proteins and this therefore needs to be accounted for. The *normalized absolute protein synthesis rate* for a gene *i* is then calculated by the following expression:
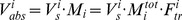
(7)where 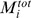 is the relative total mRNA concentration of species *i* (mRNA engaged in translation and not engaged in translation summed up), and 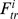 is the fraction of mRNA copies of species *i* engaged in translation (both 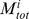 and 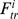 are measured experimentally [11], [17], while 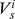 is obtained from the modeling analysis in the earlier sections).

Note that we call this rate *normalized absolute protein synthesis* rate, in the sense that we only know relative mRNA concentrations between the species and not absolute concentrations. Therefore these values are given in arbitrary units.

## Results/Discussion

The primary objective of this work was to compute the protein synthesis rates on a genome-wide scale in a prokaryote and to determine the related translational control based on the genome-wide translatome analysis. We used the ribosomal densities to estimate key kinetic parameters in a mathematical model, which was subsequently analyzed for the estimation of protein synthesis rate per mRNA and for the identification of the distribution of rate limitation between translation initiation, elongation, and termination.

We first developed a procedure to estimate the ribosomal densities from the experimental data. This procedure takes into account various factors that can influence the estimated ribosomal densities, as described in the following section. The characterization of protein synthesis and translational control are described next, and these results are then analyzed in the context of protein function. Finally, the changes resulting under stress conditions are also studied in a different experiment and we analyzed them relative to the reference conditions.

### Estimation of ribosomal density

Translatome data was obtained for *L. lactis* cells in the exponential phase for 1619 genes and their polysome sizes were assigned with confidence for 1177 genes [11]. In such experiments, a chromatogram is used to elute the mRNA copies according to their polysome sizes into different elution fractions, with help of a sucrose gradient (Figure S1). It is generally assumed that all full size mRNA copies of a given gene have in average the same polysome size, i.e. that the mRNAs of a given gene have a uniform polysome size in the cells population, and therefore they should belong to a single elution fraction or to some adjacent fractions, as is observed for most eukaryote genes [7], [10]. Some eukaryote genes had yet their mRNA copies distributed with peaks between two non-adjacent fractions. Nevertheless, such odd behavior seems to be much more common in prokaryotes, as is observed in the experimental data for *L. lactis*: the mRNA copies from many genes are distributed across all the seven elution fractions (Figure S2). The two first elution fractions, B and C, represent transcripts that are still ribosome-free (fraction B) or only in co-sedimentation with one ribosomal sub-unit (fraction C), while the other fractions are composed of mRNAs engaged in translation with average number of loaded ribosomes from 1 to 14 [11]. A distribution of the mRNA copies with multiple peaks at different polysome sizes is characteristic for many genes (Figure S2), with one peak around empty mRNA copies, one peak between polysome sizes 4.1 and 7.4 and a third peak in the last elution fraction (polysome size 14), instead of a single narrow peak as it would be expected if all the copies of the same mRNA species had the same ribosomal density.

Therefore, we first investigated the origin of these observations by testing the following five hypotheses: (i) influence of the stochasticity and intrinsic noise; (ii) effects of the partially transcribed or decaying mRNA; (iii) impact of the biophysics of co-elution; (iv) variations in the initiation process; and (v) influence of the operonic structures. In order to investigate each of these hypotheses, we analyzed the data in depth, using alternative modeling and computational approaches. These results are presented and discussed in the following subsections. Overall our analyses below suggest that the main contributors are the impact of co-elution and a modified initiation process.

#### Stochasticity

Due to the presence of low copy numbers of mRNAs in the cells and the intrinsic noise [18], [19], there can be some variability in the polysome sizes observed for a given gene. Therefore we used stochastic modeling and simulation of translation in order to examine if a similar distribution of mRNA polysome sizes is also observed. These simulations showed the polysome size of a given gene should still peak around a single value; although this peak is not necessary very narrow (see Text S1).

#### mRNA fragment size and mRNA stability

The simultaneous transcription and translation of single genes in bacteria [20] could result in incompletely transcribed mRNAs which are actively translated. Under the assumption of a uniform ribosomal density for a given mRNA species, the number of loaded ribosomes on an incomplete mRNA copy will be proportional to its length. In addition, it has been demonstrated that mRNA degradation occurred on mRNA molecules still involved in translation [21], and therefore, mRNA degradation process of full-length mRNAs will generate truncated mRNA molecules of variable sizes, which could be also actively translated by polysomes. However, when determining the polysome size of mRNAs, the experiment could not assert if the mRNA copy was a full size mRNA or only a fragment, since classic transcriptomic techniques were used.

We therefore investigated the cellular distribution of mRNA fragment sizes using a modeling approach. We developed a simplified model, where the transcription rate was similar to translation rate, and the mRNA decay of full-length mRNAs was also taken into account with mRNA half-live values as obtained experimentally [13] (see Text S1 for the model). Results revealed that more than 95% of mRNAs were full-length molecules independently of the gene length (Figure S3). Therefore, the variability of mRNA copy length could not explain the observed distribution of mRNA proportion under each polysome size. Interestingly, a recent experiment observed that co-transcriptional translation was indeed rare in *E. coli* and that only 10–15% of ribosomes were localized in the dense DNA region, where such translation of mRNA fragments could happen [22].

As an additional evidence against the possible contamination of the data due to mRNA fragments that would be present in the measurements, we observed the polysome distribution for genes grouped by their half-lives (Figure S4). We hypothesized that more stable mRNAs would stay for longer in their full-mRNA size and that thus more copies of these mRNAs could accommodate their *highest* polysome size and be engaged in translation. Surprisingly, we observed the opposite: the longer the half-life of a group was (from 2.8 to more than 18 minutes), the fewer mRNA copies it had with higher polysome sizes and the more copies it had in fractions *B* and *C* (Figure S4). This suggests that less stable mRNAs (short half-life) tend to be more engaged in translation with higher polysome sizes. This result further confirms our previous observations of the negative correlation between mRNA stabilization and translation [11], although transcript stabilization after ribosome binding is generally expected. We hypothesize that this could be a means for *L. lactis* to manage the cost of translation, with the same amount of protein produced by short-lived mRNA copies. Based on our analysis, the short-lived mRNA species are translated with larger number of ribosomes (resulting in higher synthesis rates), and the longer-lived species are translated by less ribosomes. This then implies that for short-lived mRNAs the energetic cost needed to build the mRNA is compensated by a sufficient amount of proteins produced per mRNA copy, and more stable mRNAs utilize less ribosomes as the mRNAs remain for longer time to produce the same amount of proteins. This hypothesis cannot however be assessed by such type of high-throughput experiment. In order to further confirm this observation, one should study by molecular biology approaches the link between the number of proteins synthesized per mRNA and the mRNA stability.

#### Co-elution

For 129 genes the estimated ribosomal density was very close or above the maximal theoretical ribosomal density of 1. Closer examination of the data revealed that these genes had an unexpected number of copies present in the last elution fraction. This is not possible due to the length of these genes and to their true mass, and it can cause a significant overestimation of the polysome size. Therefore we hypothesized that some mRNA copies could be co-eluted along the sucrose gradient with the heavy complexes and collected in the last fraction. This led us to recalibrate the data (as described in the Text S1), and derive the polysome size of 1'108 genes with higher confidence. This recalibration helped us eliminate some measurements that were evidently contaminated, giving stronger confidence in the data. After this, the two highest polysome size peaks observed on Figure S2 for most of the genes merged into a more likely common peak. However, this hypothesis could not explain the presence of the additional peak near fraction C in Figure S2.

#### Initiation stage and fraction C

We considered next why there exist these two separate peaks in the mRNA distribution among fractions for most gene species: one peak corresponding to mRNA copies around elution fraction C, and another peak corresponding to mRNAs bound by multiple ribosomes. We specifically hypothesized that the mRNAs in fraction C could not undergo the complete translation initiation process preventing additional ribosome loading. Indeed, fraction C would correspond to mRNA only bound by a ribosomal 50S subunit, but according to the literature on translation initiation process, binding of the large ribosome subunit alone at the initiation location is very improbable. Therefore we postulated that in fraction C, mRNAs co-sedimented with RNA binding protein complexes other than the 50S subunit, impeding translation initiation. Interestingly, and in support to this hypothesis, we also detected in fraction C the 16S rRNA specific of the small ribosome subunit. A plausible hypothesis is that the small ribosome subunit is sequestered on the mRNA by a RNA binding protein before the large ribosome subunit docking, preventing translation initiation [23]. In this sense, direct recruitment of the RNase E protein by the 30S ribosomal subunit was recently reported *in vivo* in *E. coli* [24]. This RNase (or its functional equivalent in Gram positive bacteria) is the scaffolding protein of a multi-enzyme assembly of high molecular weight (named degradosome and involved in RNA degradation) [25], [26]. Under the assumption of similar degradosome interaction with ribosomal subunit in gram positive bacteria as demonstrated in the gram negative *E. coli*, co-sedimentation of mRNA molecules with a degradosome complex in fraction C blocking translation cannot be ruled out in *L. lactis*.

To test these hypotheses, we developed a mathematical model with such initiation inhibition and found that simulations could reproduce qualitatively the observed distribution (see Text S1). Therefore, the analysis of the experimental data and our hypotheses suggest that in bacteria growing fast at their maximum growth rate, a significant fraction of mRNA is not involved in translation. Although this was really unexpected from the physiological point of view, the modeling analysis emphasized this behavior, and further experiments will be now required to understand in more detail this phenomenon.

#### Operonic structures and polysome size distribution

**Figure 2:**
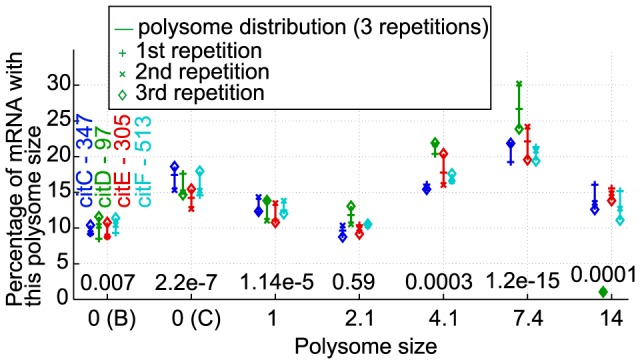
Polysome sizes of genes from experimentally verified operon *citCDEFXG*. One color was associated to each gene part of this operon (gene is absent of the figure when its experimental polysome size was missing). For each operonic gene, its mRNA proportions (from three repetitions) were plotted according to polysome size. Results from other operons are shown in Figure S5. The gene names and lengths (in number of codons) are indicated in the first fraction. The number above each polysome size is the p-value of the ANOVA test to check at a given polysome size if the mRNA proportion of the group of operonic gene is significantly different than the mRNA proportion of the rest of the genes in this fraction. For example in this figure, gene citC has a length of 347 codons, and about 10% of its mRNA copies are in fraction B, 17% in fraction C, … and 15% of its copies have a polysome size around 14; moreover, an ANOVA test shows with a p-value of 0.0001 that, in fraction of polysome size 14, the average mRNA proportion from the cit operon's genes is significantly different to the average mRNA proportion from the other gene species in this fraction (and similarly for the other elution fractions), hinting that the genes from cit operon are not distributed among fractions in the same way as the rest of the genes.

In prokaryotes, some genes are organized in operonic structure: they are transcribed under the control of the same promoter into a common mRNA molecule. We previously observed that this polycistronic structure did not induce any bias in the ribosome number determination towards high polysome size [11]. However, for some operonic genes of *L. lactis* (*argCJBF*, *argGH*, *citCDEFXG*, *optABCDF*, *trpEGDCFBA*), we examined more in details their polysome sizes (Figures 2 and S5). We found generally grouped mRNA proportions for all the genes of the same operon at a given polysome size, confirming the transcriptional link of operonic genes. Using an ANOVA test, we then checked if the mRNA proportion of the group of operonic genes in a given fraction was significantly different than the proportion of the rest of the genes (Figures 2 and S5). Depending on the operon of interest, a bias on the mRNA proportions of operonic genes was present for two to six fractions (*i.e.* operon *argGH*, with p-values for two polysome sizes 0 (B) and 2.1 lower than 0.05 (Figure S5C) or operon *citCDEFXG* with six very low p-values (<0.01, Figure 2)). Therefore, operonic structure seemed to be associated (at variable degree) with biased mRNA repartition between fractions.

This link can have an influence for the estimation of polysome sizes of genes constituent of an operon: indeed, taking as example on operon composed of two genes, A and B, where A is really occupied by 4 ribosomes, while B is occupied only by 2 ribosomes; then these 2 genes will be detected in the fraction corresponding to 6 ribosomes, in which case we might consider that both A has 6 ribosomes on it as well as B also has 6 ribosomes. Here we eventually overestimate conservatively the polysome sizes of genes part of operons; e.g. by keeping a value of 6 ribosomes in the example of the two genes. Note however that currently only 34 genes are part of known operons and 43 genes are part of hypothetical operonic structures in *L. lactis*, and for completeness we also analyzed the case where the ribosomes of these operons would have been uniformly distributed between the genes of an operon and the results did not significantly change (Figure S7).

Furthermore, the observed relation between genes of operonic structures could then be used in theory to identify putative operonic structures, in a 2-step analysis: (I) if two or more genes share the same (or very similar) mRNA distribution between elution fractions, it gives a hint that these genes might belong to a common operon; and (II) if this distribution is significantly different from the genes-averaged distribution, it gives a stronger confidence that there should exist some link (operonic or not) between these genes. The step (II) above is not a necessary condition for the genes to belong to a same operon, but it guarantees that the similar distribution of the genes observed in the step (I) is not simply coming from random luck in the choice of the genes.

### Protein synthesis rate

**Figure 3:**
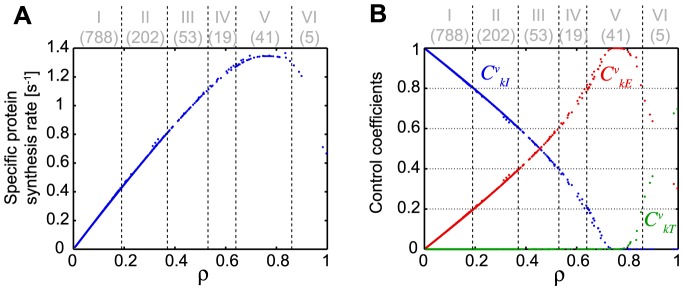
Relationship between specific protein synthesis rate, ribosomal density and control coefficients. (A) The value of the specific synthesis rate estimated for all the genes is indicated in function of the ribosomal density (obtained through the experiment). (B) The control coefficients of each translation step are shown according to ribosomal density of each gene. Initiation control coefficient 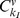, elongation control coefficient 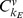 and termination control coefficient 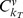 are shown. The numbers in parenthesis at the top of the figure indicate the number of genes in each group.

From the analysis of the ribosomal density (previous section), we could estimate (i) the polysome sizes of 1'108 genes (and hence their ribosomal densities), and (ii) the corresponding fraction of mRNA copies that were truly engaged in translation. We next used these two values for each gene, and a mathematical model to estimate the maximum specific protein synthesis rate for each gene (see *Materials and Methods*). The specific protein synthesis rate is defined as the number of protein molecules synthesized per second and per mRNA copy of the gene. For each gene, under the assumption of maximal synthesis rate, we determined a characteristic pair of initiation and termination rate constants that correspond to the gene's ribosomal density [15]. In the 1108 characterized *L. lactis* genes and for increasing ribosomal density the specific protein synthesis rate increased, it reached a maximum and then decreased (Figure 3A). A maximal rate of 1.3 s^−1^ (*i.e.* 1.3 molecule of protein synthesized per second and per mRNA copies) was reached for ribosomal densities between 0.7 and 0.8, for *L. lactis* cells grown at 0.88 h^−1^.

#### Characterization of translation control

Translation process is composed of three steps namely initiation, elongation and termination. The codon-specific model we use allow us to determine in a quantitative manner the rate-limiting steps of translation for each of the 1108 mRNA species [1]–[3], [15], by computing the control coefficients of initiation 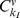, elongation 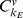 and termination 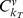, which are the sensitivities of protein synthesis rates to their respective rate constants. The initiation and termination control coefficients are defined as the percent change on synthesis rate in response to a percent change in the rate constants of initiation or termination respectively. Similarly, the elongation control coefficient is defined as the percent change of synthesis rate for a simultaneous percent change of the elongation rate constants of all codons. Note however that although for elongation the change is considered simultaneously for the rate constants of all codons, in reality, only a small number of codons contributes significantly to this overall elongation control coefficient [2], [15].

Based on mathematical analysis of the models and from the theory of sensitivity analysis and metabolic control analysis, we have shown that [1], [2]:
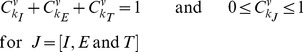
(8)The relative value of the control coefficients is then used to identify the rate limiting steps in a ranked order. For example, if 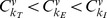, then initiation is the main rate limiting step, with elongation having higher control than termination. The control coefficients varied with the ribosomal density (Figure 3B) and for the majority of the genes (70%) the control of protein synthesis is shared between initiation and elongation (i.e. 70% of genes have at least control coefficient values above 0.05 in both initiation and elongation rate constants). Genes with low ribosomal density are predominantly initiation limited, and as the ribosomal density increases, the elongation limitation becomes dominant, reaches a value of 1, and it then decreases as the ribosomal density further approaches its maximum, where the termination control is predominant.

Highest initiation limitation (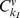
>0.8, group I in Figure 3B, S6A) was systematically associated with the lower specific protein synthesis rates and it is the most frequently observed (788 genes corresponding to 71% of the genes) (Figure 3B, Figure S6A). Interestingly, theoretical studies of the genome-wide translation in *E. coli* are in agreement with these results [1]. At the optimal protein synthesis rate (1.3 s^−1^) and ribosomal density between 0.65 and 0.85, translation rate is mainly elongation-limited (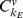
>0.8, group V), but for a smaller number of genes (41 genes corresponding to 4% of the genes) (Figure 3, Figure S6A). Moreover, in 274 genes (nearly 25% of the genes, groups II–IV) protein synthesis rate control is at least of 0.2 in both initiation and elongation simultaneously. Elongation limitation could be linked to codon usage and/or tRNA availability. In a previous computational analysis of proteomic and transcriptomic data from *L. lactis* we have shown that indeed the codon adaptation index correlated positively with estimated translation efficiencies [11], [17]. Finally, in only 5 genes, control is shared between elongation and termination.

#### Translation and protein function

**Figure 4:**
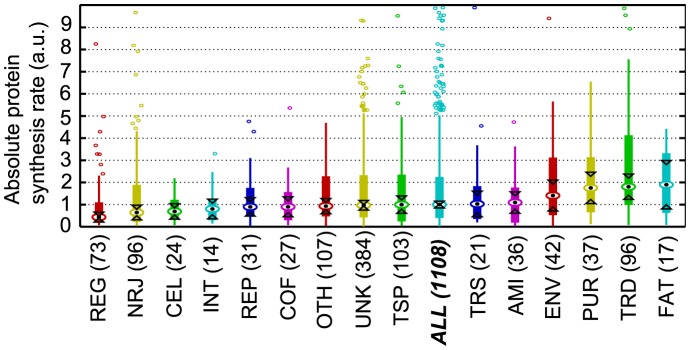
Normalized absolute protein synthesis rate by functional categories (shown as boxplots). The number of genes observed in each functional category is between brackets. Medians are symbolized by circle with a point in the middle. The boxes describe the quartiles of the data, while the lines extend to the extreme data points (not including the outliers which are shown as little open rounds). Interval endpoints are defined as the centers of the triangular markers. When the intervals of two groups do not overlap, then their medians can be assumed to be different with 95% confidence. The categories are ordered according to their median value, and the “category ALL” corresponding to all genes together is highlighted to indicate which categories perform better or worse than the cell average. AMI: amino acid biosynthesis, CEL: cellular process, COF: biosynthesis of cofactors, ENV: cell envelope, FAT: fatty acid and phospholipid metabolism, INT: central intermediary metabolism, NRJ: energy metabolism, OTH: other categories, PUR: purine, pyrimidine, nucleoside and nucleotide metabolism, REG: regulatory functions, REP: replication, TRD: translation, TRS: transcription, TSP: transport and binding proteins, UNK: unknown function.

We next examined the relationship between protein function and protein synthesis rate and control, and we compared the median absolute protein synthesis rates between *L. lactis* functional (sub)categories (Figure 4, S6B). The absolute protein synthesis rate describes the synthesis rate per gene species as opposed to the specific synthesis rate that corresponds to the synthesis rate per mRNA copy. We determined here the *normalized absolute protein synthesis rate per cell*, using the specific protein synthesis rate (analyzed in the previous sections), the relative mRNA concentration of each species with respect to total mRNA, and the fraction of mRNA copies from a gene that is actively translated (see *Materials and Methods*). To classify the proteins, we used the functional categories as defined by Bolotin et al [27], [28], as gene ontology terms do not specifically exist for *L. lactis*. Such categories are well adapted to the reduced metabolism present in this bacterium.

Genes involved in the metabolism of purines, pyrimidines, nucleosides and nucleotides (PUR), fatty acids and phospholipids (FAT), cell envelope (ENV) and also in the translation process (TRD) displayed higher protein synthesis rates than the average (Figure 4). This is consistent with the observations that proteins of the translation machinery (ribosomal proteins) are the most abundant proteins in bacteria and the observations in our previous proteomic analysis of *L. lactis* [29].

On the contrary, the translation rate of genes related to cellular process (CEL), energy metabolism (NRJ) and central intermediary metabolism (INT) was low (Figure 4). This suggests that at high growth rate, the energetic state of *L. lactis* cells is sufficient to support optimal activity of proteins (notably for energy-producing enzymes), despite a low overall protein synthesis rate of these genes. But the most striking low rate of protein synthesis was observed for genes of regulatory functions (REG). This is probably due to the fact that *L. lactis* was grown in optimal nutritional (amino acids, vitamins and other cofactors) and environmental (pH, temperature) conditions, and under such conditions the cells might not have to respond to significant stress and to translate regulatory proteins required for adaptation.

Next we performed enrichment analysis to determine if some functional categories were over-represented for some control values or synthesis rates (Tables 1, S1 and S2). We observe genes from the TRD (translation) category are among the fastest translated ones, and these genes also correspond to the ones most limited by elongation steps (Tables 1 and S2). Therefore protein translation is most sensitive to the availability of amino acids and energy, which are required for protein synthesis. We hypothesize that this can help the translation machinery to adapt the fastest to global systems changes that lead to amino acids or tRNA depletions or redistributions and to the energetic state of the cell.

**Table 1:** Functional enrichment analysis for genes grouped by similar translational control.

Group	ρ	*V_s_ [s^−1^]*	*V_abs_ [a.u.]*	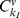	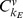	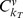	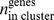	Functional category	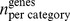
I	0.01–0.19	0.02–0.44	0.0–46.6	0.8–1	0–0.2	0	788/1108	**REG (p = 4.8·10^−4^)**	**64/73**
								**CEL (p = 2.8·10^−3^)**	**23/24**
								AMI (p = 6.8·10^−2^)	30/36
								NRJ (p = 6.9·10^−2^)	75/96
II	0.19–0.37	0.44–0.82	1.4–52.5	0.6–0.8	0.2–0.4	0	202/1108	**PUR (p = 4.3·10^−4^)**	**16/38**
								**TSP (p = 1.2·10^−2^)**	**28/103**
								**COF (p = 4.2·10^−2^)**	**9/27**
III	0.38–0.52	0.82–1.10	2.9–36.6	0.4–0.6	0.4–0.6	0	53/1108	**UNK (p = 3.9·10^−3^)**	**28/383**
								OTH (p = 6.1·10^−2^)	9/107
IV	0.53–0.64	1.11–1.28	4.2–76.3	0.2–0.4	0.6–0.8	0	19/1108	**TRD (p = 1.9·10^−2^)**	**5/96**
								UNK (p = 7.9·10^−2^)	10/383
V	0.64–0.85	1.28–1.37	3.1–121.6	0–0.2	0.8–1	0–0.2	41/1108	**OTH (p = 1.7·10^−6^)**	**15/107**
								TRD (p = 5.7·10^−2^)	7/96
VI	0.87–1.00	0.67–1.28	3.9–35.2	0	0–0.8	0.2–1	5/1108	UNK (p = 5.1·10^−2^)	4/383

Only the most enriched terms are shown, while the significantly enriched terms (p-value<0.05) are highlighted in bold. The binning is the same as on Figure 3. For each group, the ranges of ribosomal densities, specific synthesis rate, absolute synthesis rate and control coefficients are also shown. 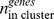 tells for example that 788 genes were present in the given cluster while 1108 genes are present in total in the data; on the other hand 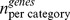 indicates for example that 64 genes of the given category were found in the cluster while 73 genes in total are present in the given category.

AMI: amino acid biosynthesis, CEL: cellular process, COF: biosynthesis of cofactors, NRJ: energy metabolism, OTH: other categories, PUR: purine, pyrimidine, nucleoside and nucleotide metabolism, REG: regulatory functions, TRD: translation, TSP: transport and binding proteins, UNK: unknown function.

On the other hand, genes under initiation control are enriched for the categories REG (regulatory functions), CEL (cellular process), AMI (amino acid biosynthesis) and NRJ (energy metabolism), while genes with both some significant elongation and initiation control mainly correspond to PUR (purine, pyrimidine, nucleoside and nucleotide metabolism), TSP (transport and binding proteins) and COF (biosynthesis of cofactors) categories (Table 1). Initiation limitations can be linked to the availability of initiation factors and ribosomes; therefore, as it was observed in bacteria that the number of ribosomes correlates with cell growth rate [30], we can speculate that such initiation limitation forces genes from these categories to adapt their synthesis rate to cellular growth rate, making the overall content needed by the cell to grow remains in proportion to the cell needs. Interestingly we observe that despite having a higher translation initiation control and lower specific synthesis rate, some of these genes from groups I and II still display a high absolute protein synthesis rate. For example, genes from PUR category are mostly enriched with a high initiation control, but these genes still display high absolute protein synthesis rates (Table 1, Figure 4). These properties could allow the cells to achieve a high production of certain proteins, while keeping them insensitive to translational limitations. Indeed the categories found enriched in groups I and II correspond to some essential elements for the cell (for example AMI, CEL, COF, NRJ, PUR) and having a limited elongation control for these genes could guarantee some constant basal synthesis rate of these proteins.

Remarkably, when observing enrichment in functional sub-categories (Table S1), it emerges that translation related genes are split among different controls: TRDdeg (degradation of proteins, peptides and glycopeptides) are found among the mostly initiation-limited genes suggesting that degradation is not impacted by translation elongation limitations, while genes of TRDfac (translation factors) and TRDsyn (ribosomal proteins synthesis and modifications) will be the most affected in case of translation limitations, suggesting that the cells do not spend significant amount of energy in building proteins that could not be used efficiently.

In recent studies [1], [31]–[34] the idea of a single rate-limited step of translation, mainly via the initiation process, is abandoned and a fine-tuning of translation control, via mixed initiation and elongation control, is proposed. Our results highlighted that indeed several combinations of translation controls were present in bacteria. Our analysis further supports a close relationship between kinetic control and protein function as a mechanism to ensure protein synthesis adjustment to cellular states and demands.

### A stressed *L. lactis*

In order to characterize the changes occurring at the translatome level under stress conditions and at reduced growth rate, we analyzed an experiment where *L. lactis* cells were challenged with depletion of isoleucine and the translatome studied on the genome-scale [35].

A recalibration of the data was first performed, as done earlier for the normal conditions (see Text S1), resulting in a total of 1405 characterized genes. Interestingly we observed that in the stress condition a higher proportion of genes was mainly under elongation control or under control shared between elongation and initiation (Figure S6C). Under these conditions, very few genes also appeared to be under termination control. As this experiment was performed under isoleucine depletion, we hypothesize that this observed increased elongation control is mainly due to an increased control from isoleucine codons, where ribosomes are probably forming queues along the mRNA.

**Figure 5:**
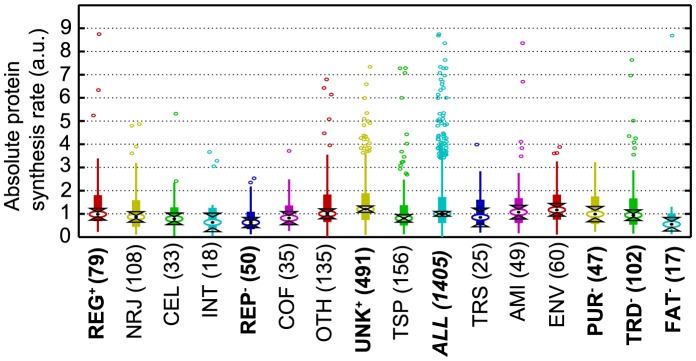
*L. lactis* under stress. This is the same as Figure 4, but for cells grown under stress condition. The ordering of categories is the same as for Figure 4; categories whose synthesis rate changed significantly between the conditions are marked in bold, with a “+” to indicate an increase and a “−” for a decrease.

Comparing the absolute protein synthesis rates grouped by (sub)categories with those under normal conditions (Figure 4, 5 and S6), it emerges that stress conditions cause a decrease in the rates of several biogenesis-related functional categories (FAT, REP, PUR and TRD), which is in agreement with the reduction of growth. For most of these functional categories (*i.e.* FAT, PUR and TRD) a decrease of protein levels was previously observed when growth rate is reduced [17]. It should however be underlined that stressed *L. lactis* cells were still metabolically active with low but significant glucose consumption and lactate production rates (Figure S1). Interestingly, we observed that the genes for the regulatory category (REG) increased synthesis rate in comparison to their value under the normal conditions, probably due to a need of the cells to cope with the stress conditions. This result about the key role of regulatory functions in adaptation is also consistent with the high protein level measured in the regulatory category during starvation in earlier studies [29]. Additionally, genes of the subcategory AMIbba (for branched chain amino acid biosynthesis) had an increased absolute synthesis rate relative to the normal conditions (Figure S6B and S6D). This result is supported experimentally by the increase of *in vivo* protein concentration for two proteins IlvD and LeuC of the isoleucine biosynthetic pathway (respectively, 4-fold and 2.5-fold in stress compared to normal conditions) [12]. *De novo* isoleucine synthesis was therefore metabolically active and allowed *L. lactis* to survive and even to grow in the total depletion of isoleucine in the growth medium. Consistently, the other amino acid biosynthetic pathways did not show any specific increase in synthesis rate as they were supplied by the growth medium.

### Conclusion

In this paper, we developed a novel strategy to analyze translatomics data in bacteria, allowing robust estimation of polysome size and ribosome occupancies at the genome-scale level. We used a mechanistic model of protein synthesis to analyze the translatome in *L. lactis* cells in exponential phase and also under stress condition using experimentally determined ribosomal densities. Other groups have previously studied translatome in eukaryotic systems, with help of various techniques (e.g. with polysome gradients similar to the data used here; or with help of the novel technique of ribosome profiling, which asses the position of ribosomes on the mRNA but does not strictly measure the polysome size, see also below for discussion) [7], [8], [16], [36], [37]. Additionally the ribosome profiling method was recently applied to prokaryotes systems in order to get information on the positioning of ribosomes along the mRNA [38], [39]. Nevertheless, this is the first study of polysome gradients for prokaryotic cells. In order to resolve some important issues in the quantification of ribosomal density in prokaryotes, we first developed a method for data preprocessing and analysis to better determine the ribosomal density.

The analysis has further estimated protein synthesis rates at the genome scale level and deciphered the key regulatory steps for translation. Analysis of the model allowed us to identify the rate limiting steps for protein synthesis of each gene. We specifically quantified the distribution of control between initiation, elongation, and termination, and our results support the concept of *mixed* control of translation in bacteria, in the particular case of *L. lactis*. Contrary to general belief, it emerges from the study that translation elongation has a significant impact on a large proportion of genes. Most of the genes are under initiation and elongation control, and fewer are under elongation and termination control.

In addition, we have provided new results on the link between translation regulation and metabolism. Functional enrichment analysis suggests that genes with similar function share common synthesis rate and rate limitation properties. In *L. lactis*, genome-scale translation regulation was used to adapt the metabolic network to growth conditions. In both optimal and stress environments, particular metabolic pathways were more or less favored by regulating protein synthesis rates. Furthermore, depending on gene function, the protein synthesis rate was controlled by the nature of the rate-limiting step to be sensitive or not to translational limitations. In particular, we identified main differences between the regulation of various functional categories: the genes affected most by perturbation on elongation rates were those related to translation, probably enabling a fast redistribution of translation component when needed, while genes with stronger initiation control were related to some essential elements for the cell (AMI, CEL, COF, NRJ, PUR), possibly ensuring that these proteins keep an abundance independent of elongation perturbations.

Comparative, model-based analysis of the translatome under different physiological states (optimal growth conditions vs. amino-acid starvation) provided significant insights on the specific role of protein synthesis, for groups of proteins and individual proteins. We observed a redistribution of protein synthesis rate and control limitations for some functional categories, and mainly for regulatory proteins.

Overall, the methods and the analysis procedure developed here is general and it can serve as a reference procedure for translatome analyses of other prokaryotic systems. Our findings about the importance of the elongation in translation control suggests that it is important to further characterize the position of the ribosomes along the mRNA in addition to the number of ribosomes per chain. If ribosomes are queuing near specific codons, this might cause additional redistribution of the elongation control along the mRNA sequence, with important implications. In order to measure such positioning of ribosomes along the mRNA sequence, one could build an experiment similar to the *ribosome density mapping* developed by Arava et al. in *Saccharomyces cerevisiae* [8]. There they measured the polysome sizes of fragments of mRNA after cutting the mRNA sequences in two to three fragments, and they observed that the ribosome distribution stayed uniform over the whole gene. Such an experiment could then support the assumptions of the present model, where termination rate constant is assumed non-limiting for most genes and elongation rate constant is approximated as uniform along the full sequence: for example if the termination rate constant would be limiting, then such an experiment would observe a queuing of ribosomes near of the stop codon. By cutting the mRNAs near of the start codon, one could additionally assess the validity of considering that all the ribosomes are active in translation, or in contrary observe that some ribosomes are bound in the 5′ UTR.

The positioning of ribosomes along the mRNA sequence could also be measured, with the ribosome profiling strategy first presented in *S. cerevisiae* [16], which was subsequently also applied to *E. coli* and *B. subtilis* [38], [39]. Note however the major difference between ribosome profiling and polysome gradients: with ribosome profiling, it is possible to estimate the position of ribosomes on mRNA, however one cannot tell if the ribosomes are coming from the same mRNA copy or from different copies (i.e. the method is not able to tell if it is one mRNA copy that had 10 ribosomes and a second copy of the same gene that was empty, or if the two mRNA copies had 5 ribosomes each for example), and in the analysis presented in the present study, it is exactly such information on the number of ribosomes on each mRNA that was needed, which is exactly what the polysome gradients gives.

In order to further assess the power of the presented framework, one could also build an additional experiment that would measure the *in vivo* synthesis rate of some labeled protein species and measure *simultaneously* the polysome sizes of their mRNA to observe if there is a good concordance between the simulations and measurements.

## Supporting Information

Figure S1 **Schematic overview of biological data collection.** This scheme presents the different steps from bacterial culture to translatome procedure to experimentally determine the number of bound ribosomes on each mRNA molecule. The top sub-figure and table show the kinetic profiles and parameters of *L. lactis* batch culture; (1) and (2) refer to the two sampling conditions: (1) in normal condition during exponential growth (0.88 h^−1^) at 2.75 hours of culture and (2) in stress condition under isoleucine depletion at 6.25 hours of culture corresponding to a low growth rate (0.05 h^−1^). The bottom sub-figures describe the different steps of the translatome experiment, with first the size fractionation of mRNA-ribosome complexes on sucrose gradient. A typical polysomal profile obtained with a sample taken in normal condition is provided. mRNA-ribosome complexes were separated in seven fractions B to H: B and C represent transcripts that are ribosome-free (fraction B) or only in co-sedimentation with the large ribosomal sub-unit (fraction C), while the other fractions are composed of mRNAs engaged in translation. The seven fractions were subsequently hybridized to microarrays for mRNA quantification. For each microarray series, a statistical treatment of the data was performed to determine the number of bound ribosomes on each mRNA molecules.
(PDF)

Figure S2 **mRNA proportions as a function of polysome size for 15 randomly chosen genes (among a set of 1619 genes).** Each colored line stands for the distribution of a different gene among the 7 elution fractions. For each gene, the average value between triplicate measurements is used. The written polysome size corresponds to the average polysome size of the elution fractions (values 0 (B and C) are for empty mRNA or mRNA for which a complete ribosome is not bound).
(PDF)

Figure S3 **Distribution of percentages of mRNA copies with a given size according to the proportion of non full length.** Each color represents a gene length ranging from 97 to 311 codons. These results were obtained from simulations as described in the Text S1.
(PDF)

Figure S4 **Relationships between polysome size and mRNA stability.** Means ± standard deviations of mRNA proportions were plotted according to polysome size, mRNAs being grouped (205 mRNAs per group) according to their half-life value (one color per group). The two first “polysome sizes”, 0 (B and C) correspond to the mRNA observed in fractions B and C that do not have a full ribosome bound to them. HL: half-life.
(PDF)

Figure S5 **Polysome sizes of operonic genes.** Similar to Figure 2, the distribution of mRNA copies between elution fractions for operonic genes is shown (see Figure 2 for details). Results for the following operons are shown: (A) *trpEGDCFBA*; (B) *optABCDF*; (C) *argGH*; (D) *argCJBF*. (A) is an experimentally verified operon, while (B–D) are hypothetical operons.
(PDF)

Figure S6 **Comparing results from normal and stress conditions.** (A–B) are obtained from the experiments with optimal growth condition, and (C–D) are under isoleucine starvation condition. (A and C) show histogram of the specific protein synthesis rate, grouped by translation limitations (the bins are defined in Figure 3). Note that the specific synthesis rate for (C) is in arbitrary units, because in the stress condition, the data necessary to compute an average translation elongation rate were not measured. (B and D) present normalized absolute protein synthesis rates for the subcategories of AMI (see Figure 4 and 5 for description of the plot). AMIaro: aromatic amino acid family , AMIasp: aspartate family , AMIbba: branched chain family , AMIglu: glutamate family, AMIhis: histidine family, AMIser: serine.
(PDF)

Figure S7 **Same as** **Figure 3** **and Figure S6A, but considering the polysome size of operonic genes is shared between these genes, as explained in the text.** (A) Synthesis rate and (B) Control coefficients for initiation, termination and elongation rate constants are shown in function of the ribosomal density of the genes; (C) histograms of the specific protein synthesis rate obtained in this condition.
(PDF)

Figure S8 **Distribution of mRNA copies between polysome sizes obtained from stochastic simulations.** For each case, 3 repetitions of the stochastic simulations are shown (different colors), and each subfigure represents the results obtained with different values for the translation initiation and termination rate constants: (A) lower initiation rate constant and high termination rate constant; (B) medium initiation rate constant, high termination rate constant; (C) high initiation rate constant, medium termination rate constant.
(PDF)

Figure S9 **Simulations with an initiation-inhibiting complex.** (A) scheme used to model the binding of a complex inhibiting the 70S initiation: when the inhibiting complex is bound, then no further translation initiation can happen as long as this complex is bound; and once a complete 70S has been initiated, then the translation goes on with the usual steps of elongation (*R* are the free ribosomes; *N* the inhibition complex; and *k_j_* the various rate constants). (B–D) mRNA copies distributions obtained from stochastic simulations with various rates of translation initiation and rates of binding and unbinding for the inhibiting complex.
(PDF)

Table S1 **Enrichment of functional sub-categories for genes grouped by similar translational control.** See Table 1 for a description, where here we look for terms of functional sub-categories.
(PDF)

Table S2 **Functional enrichment for genes with highest or lowest specific or absolute protein synthesis rate.**
(PDF)

Text S1 **Supplementary methods.**
(PDF)
