# Supplementary material for: A Genome-Scale Integration and Analysis of Lactococcus lactis Translation Data
Source: PLoS Comput Biol. 2013 Oct 10;9(10):e1003240. doi: 10.1371/journal.pcbi.1003240 (PMC3794899; doi:10.1371/journal.pcbi.1003240)

Batch culture of *L. lactis*

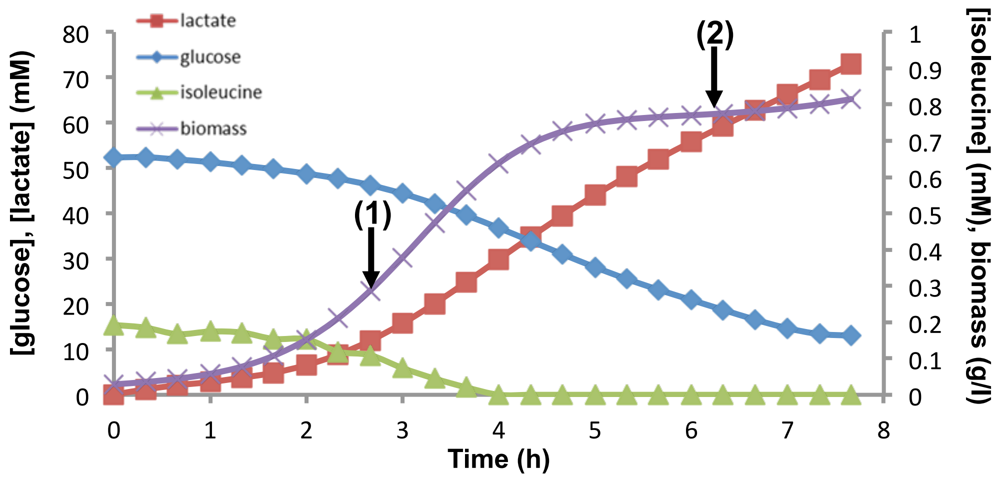

Sampling condition	Growth rate (h^{-1})	$q_{glucose}$ ($mmol.g^{-1}.h^{-1}$)	$n_{lactate}$ ($mmol.g^{-1}.h^{-1}$)
(1) normal	0.88	22 ± 3	42 ± 2
(2) stress : isoleucine depletion	0.05	7 ± 1	15 ± 1

Translation arrest with chloramphenicol and sampling

Size separation of ribosome-mRNA complexes according to ribosome number

(Example of a polysomal profile in normal condition (1))

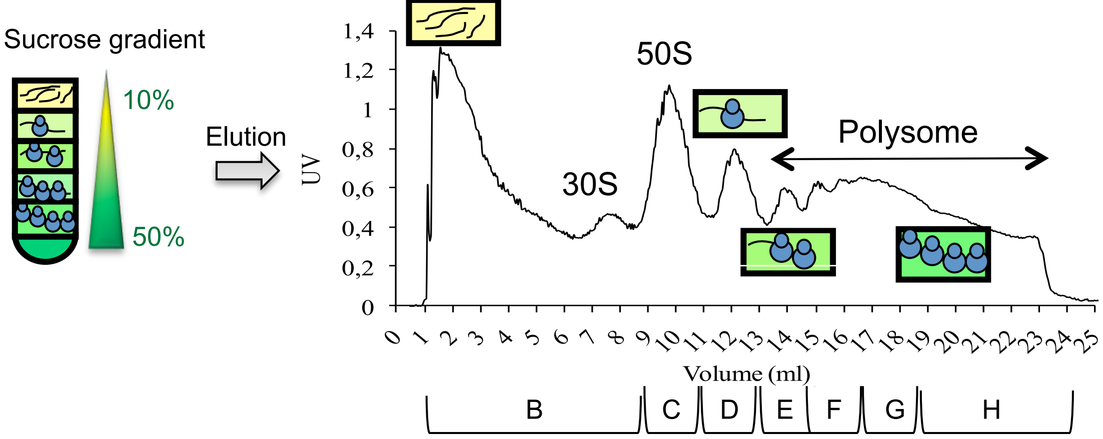

Quantification of mRNA levels in each fraction by microarrays

Statistical data processing

Determination of the number of ribosomes bound to each mRNA molecules

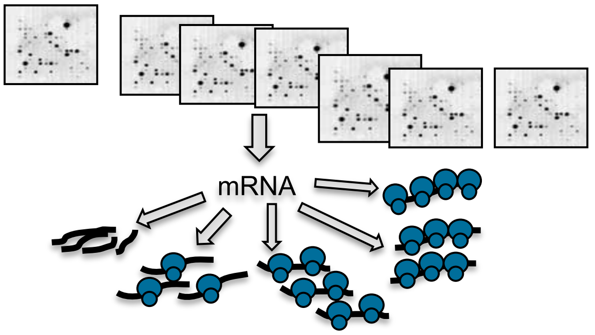

Supplement: Figure S1 — Schematic overview of biological data collection. This scheme presents the different steps from bacterial culture to translatome procedure to experimentally determine the number of bound ribosomes on each mRNA molecule. The top sub-figure and table show the kinetic profiles and parameters of L. lactis batch culture; (1) and (2) refer to the two sampling conditions: (1) in normal condition during exponential growth (0.88 h−1) at 2.75 hours of culture and (2) in stress condition under isoleucine depletion at 6.25 hours of culture corresponding to a low growth rate (0.05 h−1). The bottom sub-figures describe the different steps of the translatome experiment, with first the size fractionation of mRNA-ribosome complexes on sucrose gradient. A typical polysomal profile obtained with a sample taken in normal condition is provided. mRNA-ribosome complexes were separated in seven fractions B to H: B and C represent transcripts that are ribosome-free (fraction B) or only in co-sedimentation with the large ribosomal sub-unit (fraction C), while the other fractions are composed of mRNAs engaged in translation. The seven fractions were subsequently hybridized to microarrays for mRNA quantification. For each microarray series, a statistical treatment of the data was performed to determine the number of bound ribosomes on each mRNA molecules. (PDF) [file pcbi.1003240.s001.pdf]
