# Supplementary figures and images for: A Genome-Scale Integration and Analysis of Lactococcus lactis Translation Data
Source: PLoS Comput Biol. 2013 Oct 10;9(10):e1003240. doi: 10.1371/journal.pcbi.1003240 (PMC3794899; doi:10.1371/journal.pcbi.1003240)

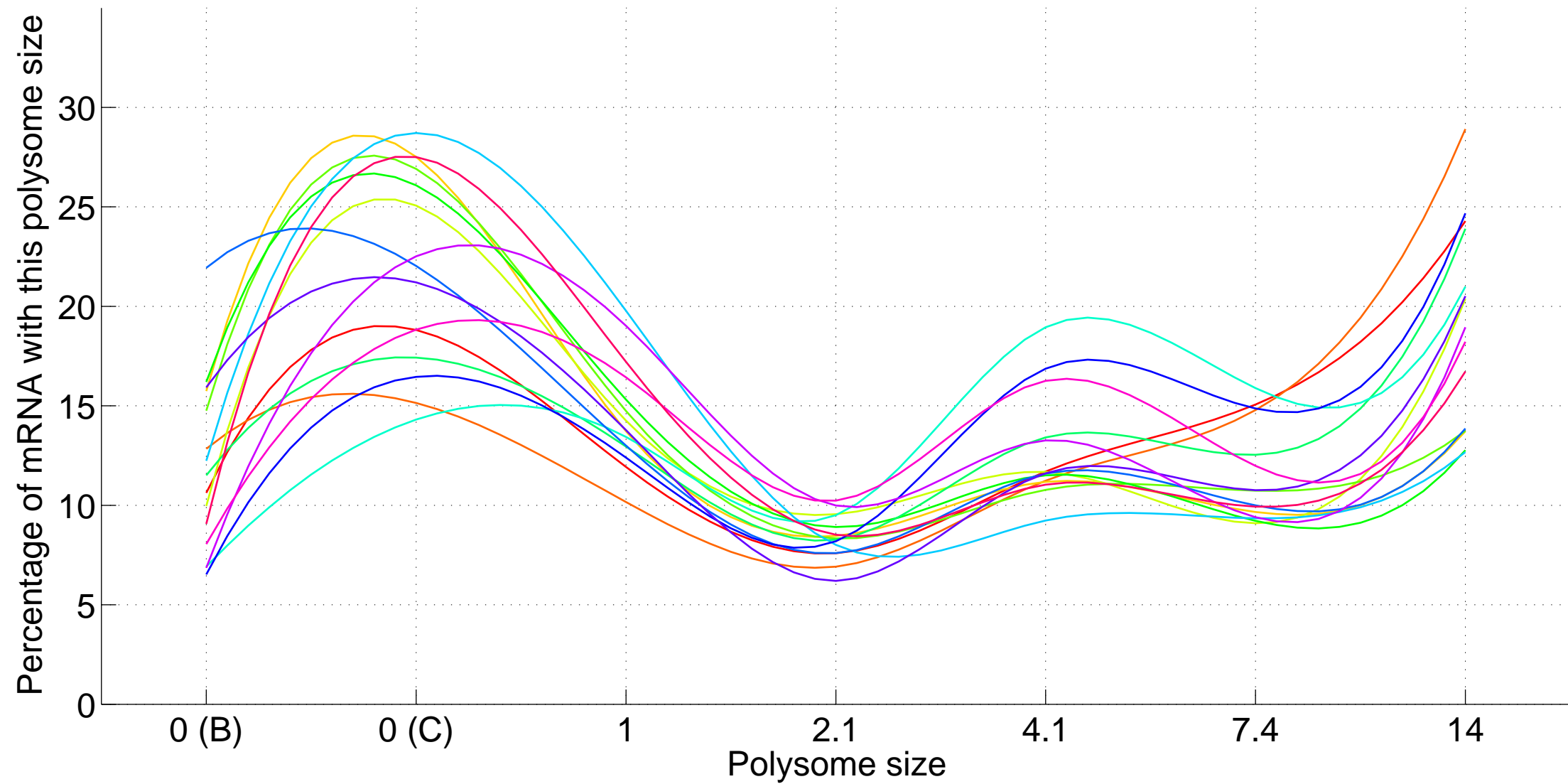

Supplement: Figure S2 — mRNA proportions as a function of polysome size for 15 randomly chosen genes (among a set of 1619 genes). Each colored line stands for the distribution of a different gene among the 7 elution fractions. For each gene, the average value between triplicate measurements is used. The written polysome size corresponds to the average polysome size of the elution fractions (values 0 (B and C) are for empty mRNA or mRNA for which a complete ribosome is not bound). (PDF) [file pcbi.1003240.s002.pdf]

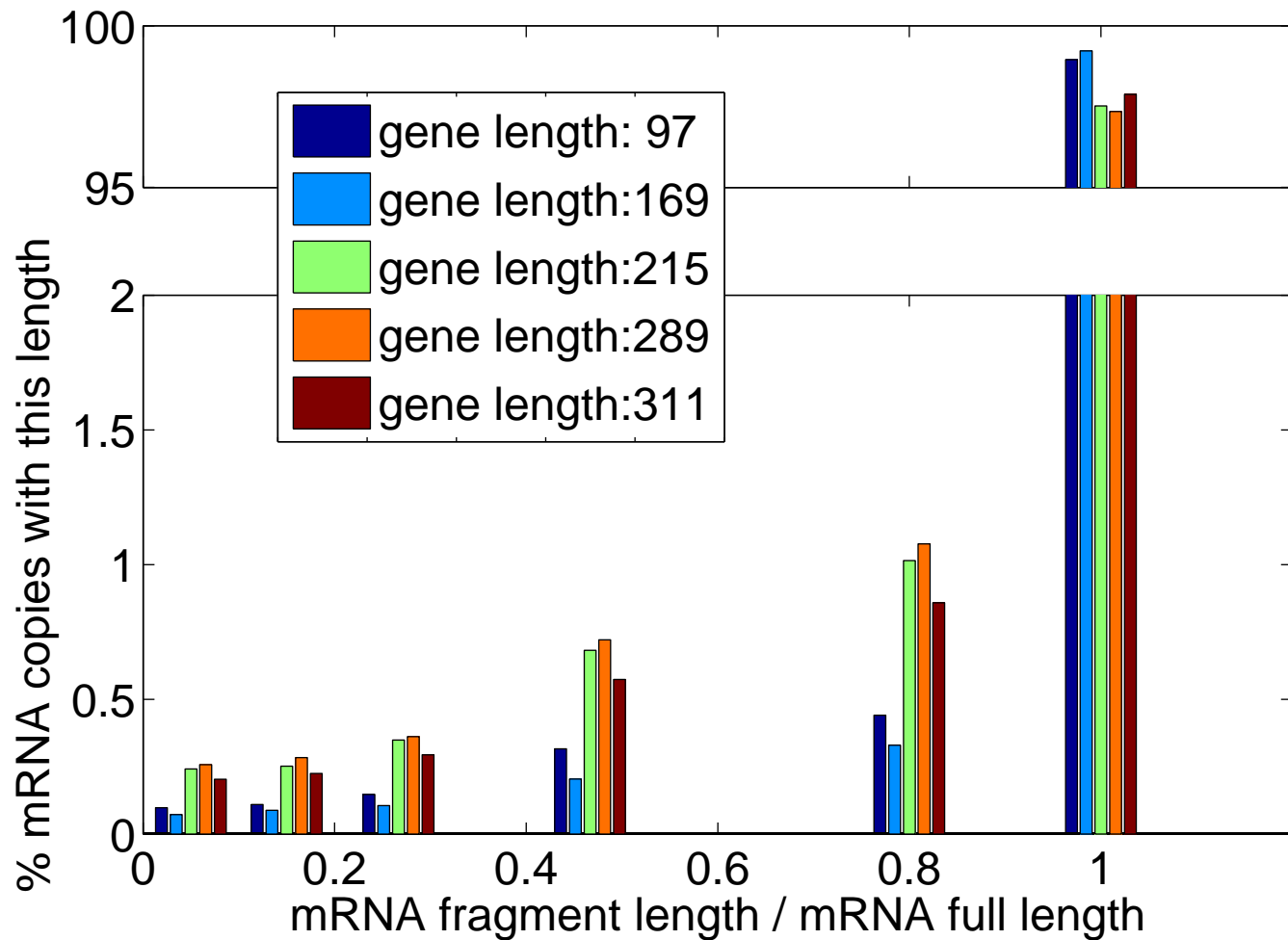

Supplement: Figure S3 — Distribution of percentages of mRNA copies with a given size according to the proportion of non full length. Each color represents a gene length ranging from 97 to 311 codons. These results were obtained from simulations as described in the Text S1. (PDF) [file pcbi.1003240.s003.pdf]

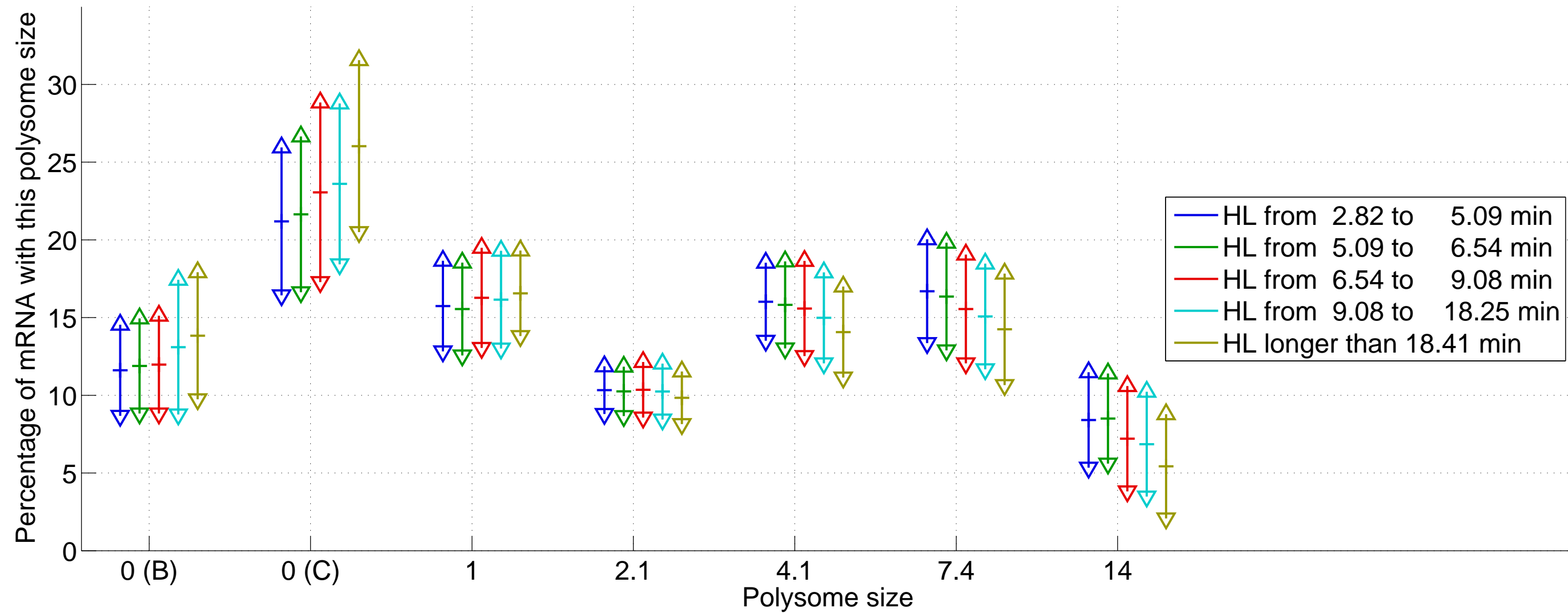

Supplement: Figure S4 — Relationships between polysome size and mRNA stability. Means ± standard deviations of mRNA proportions were plotted according to polysome size, mRNAs being grouped (205 mRNAs per group) according to their half-life value (one color per group). The two first “polysome sizes”, 0 (B and C) correspond to the mRNA observed in fractions B and C that do not have a full ribosome bound to them. HL: half-life. (PDF) [file pcbi.1003240.s004.pdf]

A

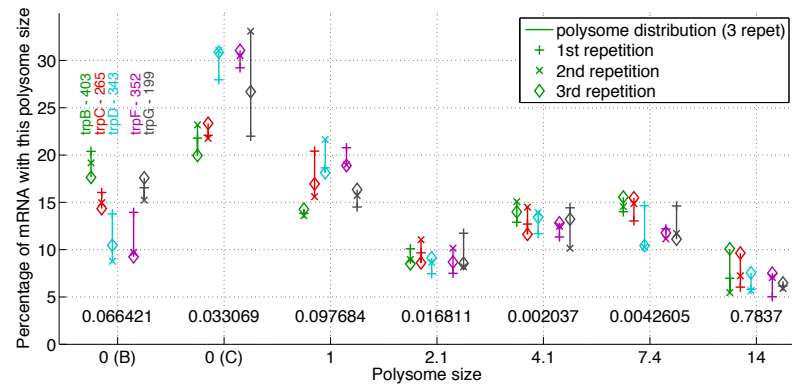

B

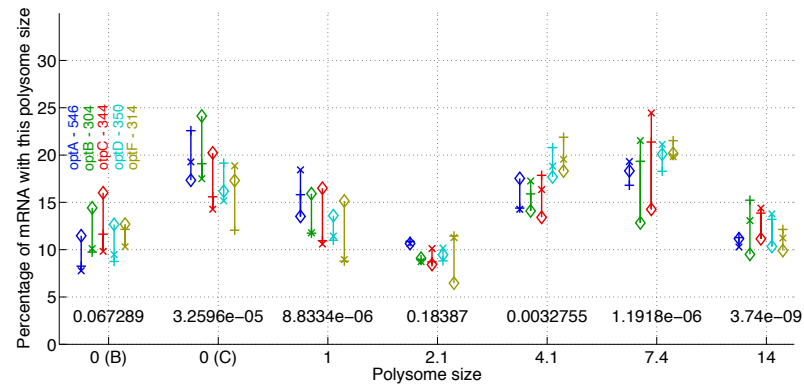

C

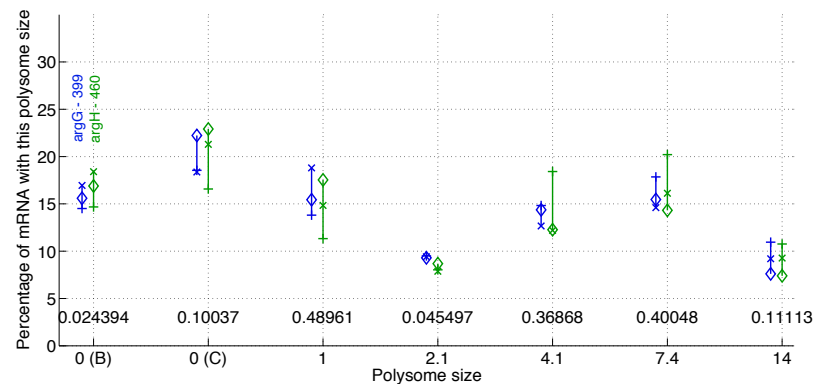

D

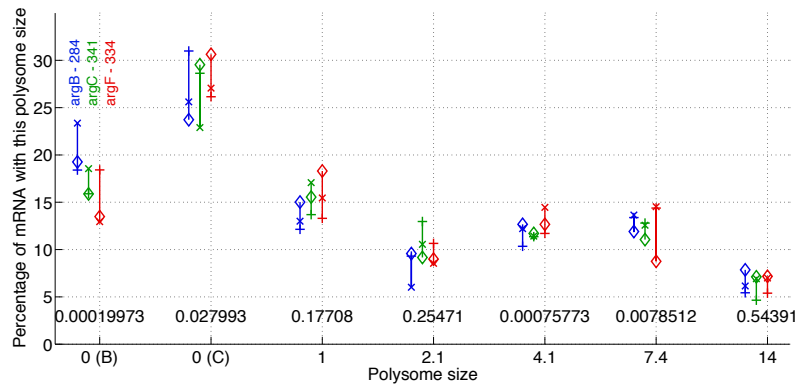

Supplement: Figure S5 — Polysome sizes of operonic genes. Similar to Figure 2, the distribution of mRNA copies between elution fractions for operonic genes is shown (see Figure 2 for details). Results for the following operons are shown: (A) trpEGDCFBA; (B) optABCDF; (C) argGH; (D) argCJBF. (A) is an experimentally verified operon, while (B–D) are hypothetical operons. (PDF) [file pcbi.1003240.s005.pdf]

A

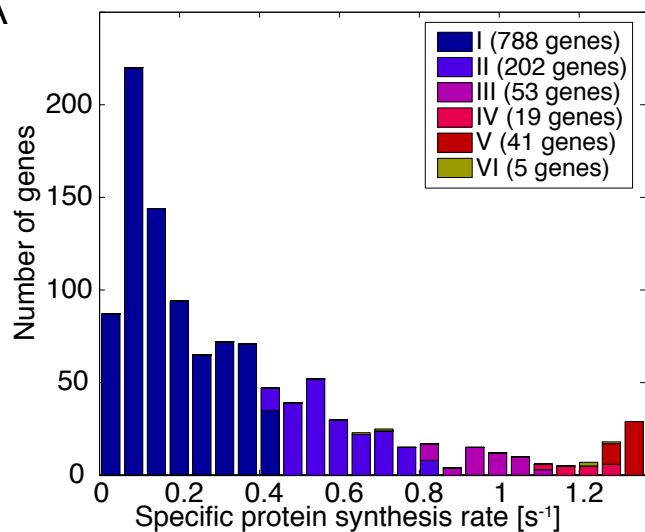

B

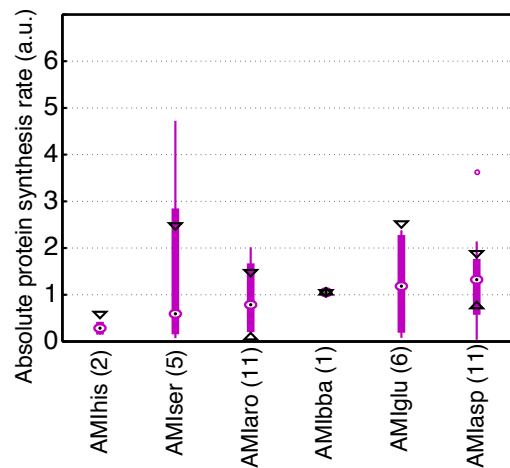

C

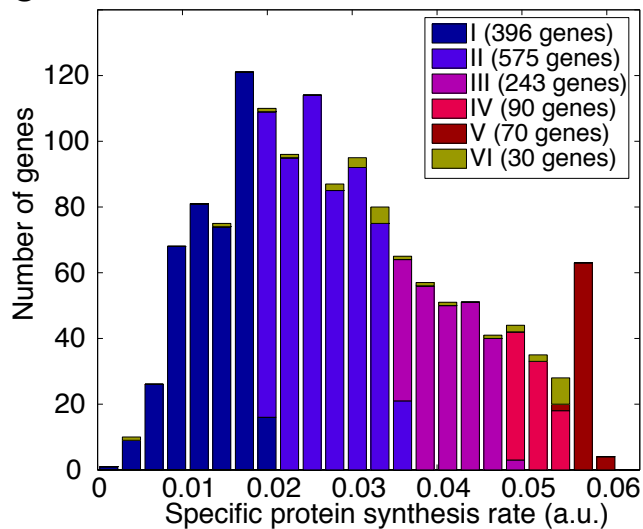

D

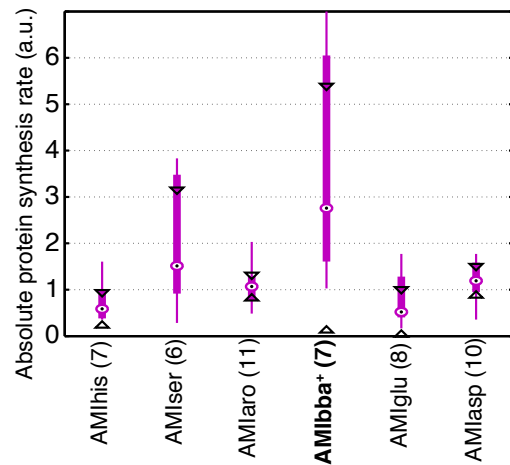

Supplement: Figure S6 — Comparing results from normal and stress conditions. (A–B) are obtained from the experiments with optimal growth condition, and (C–D) are under isoleucine starvation condition. (A and C) show histogram of the specific protein synthesis rate, grouped by translation limitations (the bins are defined in Figure 3). Note that the specific synthesis rate for (C) is in arbitrary units, because in the stress condition, the data necessary to compute an average translation elongation rate were not measured. (B and D) present normalized absolute protein synthesis rates for the subcategories of AMI (see Figure 4 and 5 for description of the plot). AMIaro: aromatic amino acid family , AMIasp: aspartate family , AMIbba: branched chain family , AMIglu: glutamate family, AMIhis: histidine family, AMIser: serine. (PDF) [file pcbi.1003240.s006.pdf]

A

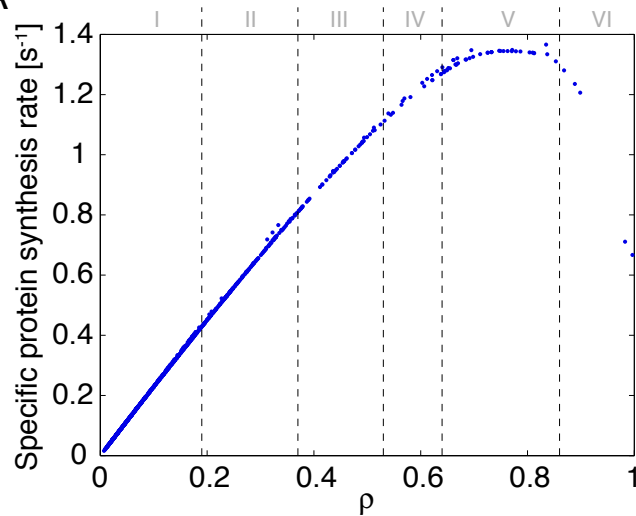

B

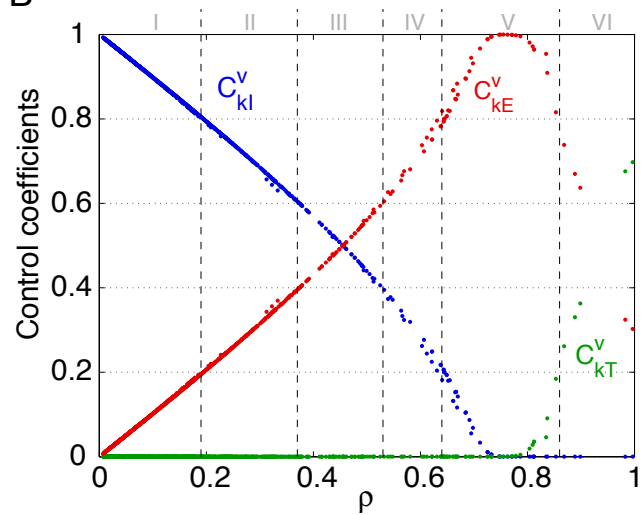

C

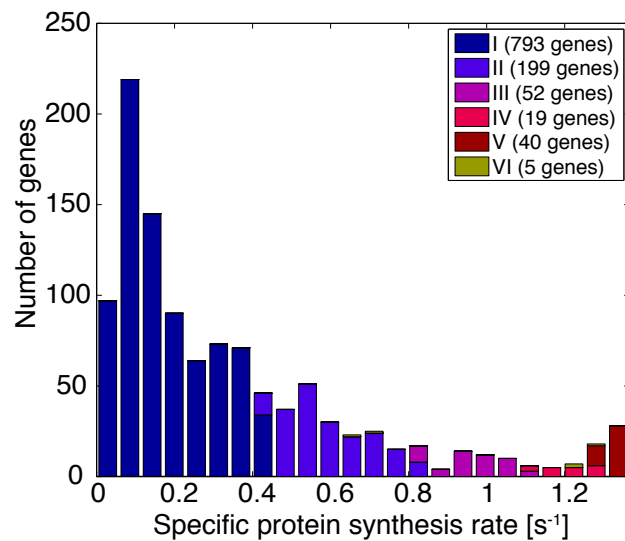

Supplement: Figure S7 — Same as Figure 3 and Figure S6A, but considering the polysome size of operonic genes is shared between these genes, as explained in the text. (A) Synthesis rate and (B) Control coefficients for initiation, termination and elongation rate constants are shown in function of the ribosomal density of the genes; (C) histograms of the specific protein synthesis rate obtained in this condition. (PDF) [file pcbi.1003240.s007.pdf]

A

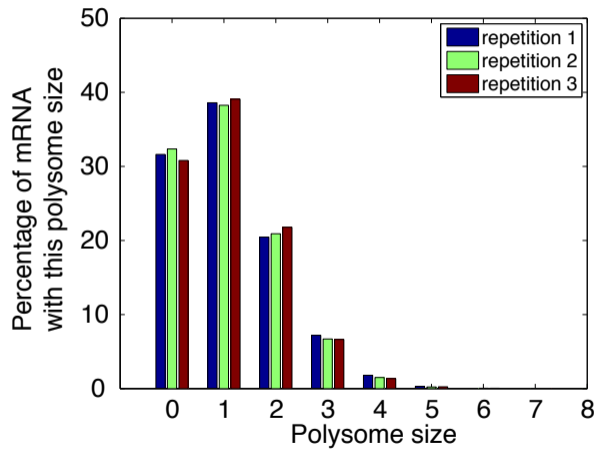

B

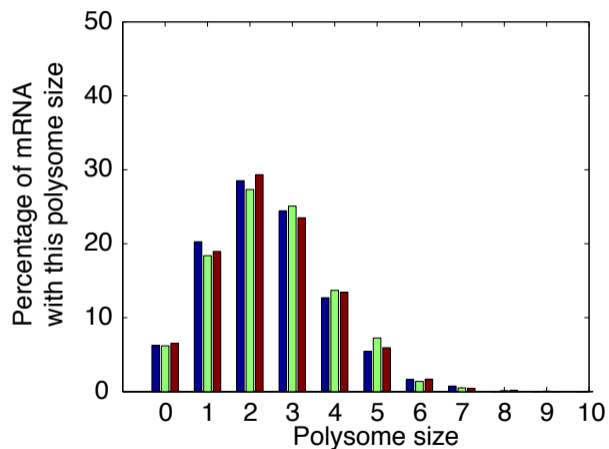

C

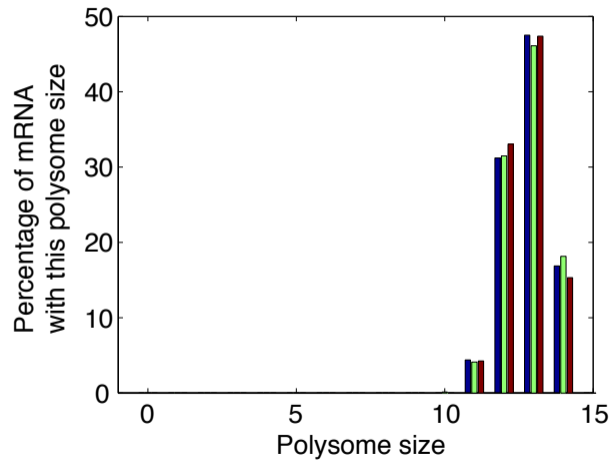

Supplement: Figure S8 — Distribution of mRNA copies between polysome sizes obtained from stochastic simulations. For each case, 3 repetitions of the stochastic simulations are shown (different colors), and each subfigure represents the results obtained with different values for the translation initiation and termination rate constants: (A) lower initiation rate constant and high termination rate constant; (B) medium initiation rate constant, high termination rate constant; (C) high initiation rate constant, medium termination rate constant. (PDF) [file pcbi.1003240.s008.pdf]

A

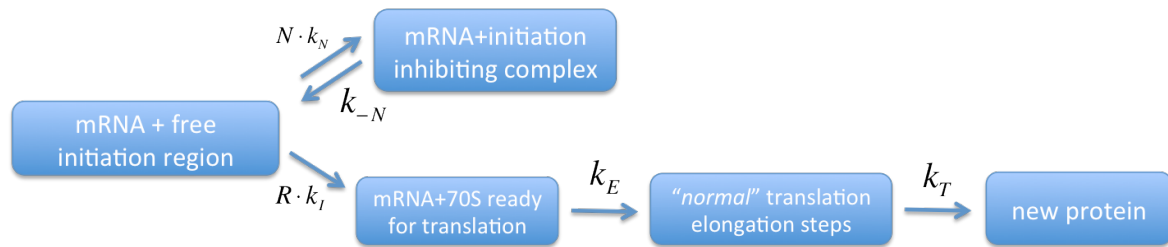

B

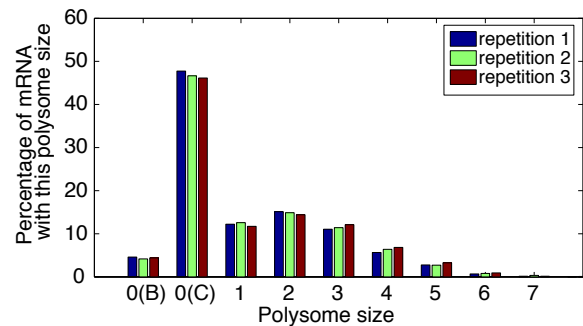

C

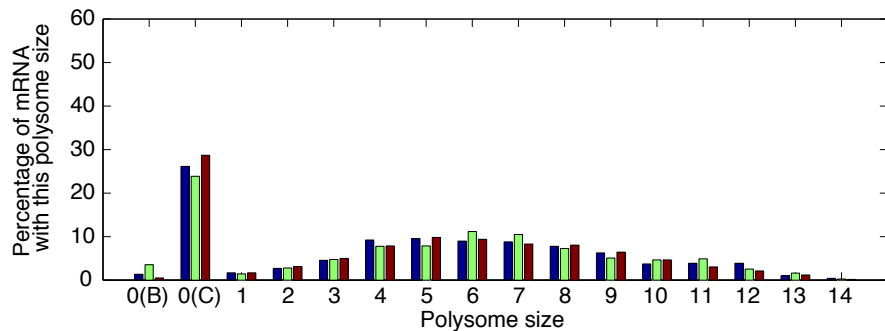

D

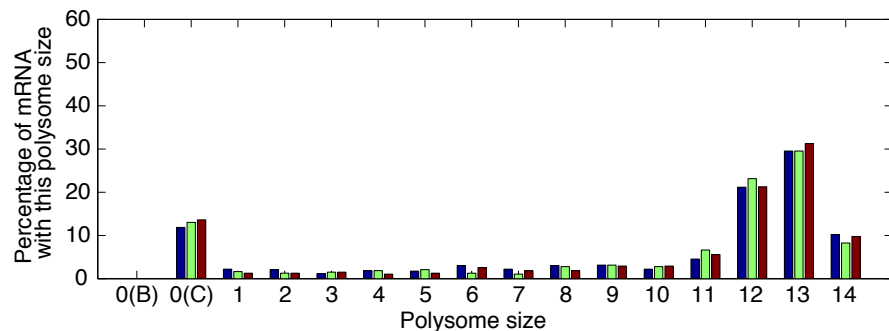

Supplement: Figure S9 — Simulations with an initiation-inhibiting complex. (A) scheme used to model the binding of a complex inhibiting the 70S initiation: when the inhibiting complex is bound, then no further translation initiation can happen as long as this complex is bound; and once a complete 70S has been initiated, then the translation goes on with the usual steps of elongation (R are the free ribosomes; N the inhibition complex; and kj the various rate constants). (B–D) mRNA copies distributions obtained from stochastic simulations with various rates of translation initiation and rates of binding and unbinding for the inhibiting complex. (PDF) [file pcbi.1003240.s009.pdf]
