# Supplementary material for: A Genome-Scale Integration and Analysis of Lactococcus lactis Translation Data
Source: PLoS Comput Biol. 2013 Oct 10;9(10):e1003240. doi: 10.1371/journal.pcbi.1003240 (PMC3794899; doi:10.1371/journal.pcbi.1003240)
Supplement: Table S1 — Enrichment of functional sub-categories for genes grouped by similar translational control. See Table 1 for a description, where here we look for terms of functional sub-categories. (PDF) [file pcbi.1003240.s010.pdf]

Group	ρ	V_s [s^{-1}]	V_{abs} [a.u.]	$C^{v}_{k_I}$	$C^{v}_{k_E}$	$C^{v}_{k_T}$	$n^{\text{genes}}_{\text{in cluster}}$	Functional sub-category	$n^{\text{genes}}_{\text{per category}}$
I	0.0 - 0.2	0.02 - 0.44	0.0 - 46.6	0.8 - 1.0	0 - 0.2	0	788/1108	**REGgen (p=2.8·10^{-2})**	**31/36**
								**NRJsug (p=3.1·10^{-2})**	**15/16**
								CELcom (p=9.1·10^{-2})	7/7
								NRJfer (p=9.1·10^{-2})	7/7
								TRDdeg (p=9.7·10^{-2})	11/12
II	0.2 - 0.4	0.44 - 0.82	1.4 - 52.5	0.6 - 0.8	0.2 - 0.4	0	202/1108	**TSPani (p=2.9·10^{-3})**	**5/7**
								**PURpyr (p=7.0·10^{-3})**	**6/11**
								**PURsal (p=7.0·10^{-3})**	**6/11**
								**COFhem (p=3.3·10^{-2})**	**2/2**
								TRDsyn (p=5.1·10^{-2})	15/54
								TSPami (p=5.5·10^{-2})	7/20
								REGdeo (p=8.7·10^{-2})	2/3
III	0.4 - 0.5	0.82 - 1.10	2.9 - 36.6	0.4 - 0.6	0.4 - 0.6	0	53/1108	**UNK (p=3.9·10^{-3})**	**28/383**
								**NRJatp (p=2.0·10^{-2})**	**2/5**
IV	0.53 - 0.64	1.11 - 1.28	4.2 - 76.3	0.2 - 0.4	0.6 - 0.8	0	19/1108	**TRDfac (p=7.3·10^{-3})**	**2/8**
								TRDsyn (p=6.1·10^{-2})	3/54
								UNK (p=7.9·10^{-2})	10/383
V	0.64 - 0.85	1.28 - 1.37	3.1 - 121.6	0 - 0.2	0.8 - 1.0	0 - 0.2	41/1108	**OTHpha (p=7.5·10^{-7})**	**14/87**
								**TRDsyn (p=2.7·10^{-3})**	**7/54**
VI	0.87 - 1.00	0.67 - 1.28	3.9 - 35.2	0	0 - 0.8	0.2 - 1	5/1108	UNK (p=5.1·10^{-2})	4/383

Table S1: Enrichment of functional sub-categories for genes grouped by similar translational control. See Table 1 for a description, where here we look for terms of functional sub-categories.

CELcom: cellular processes, transformation; COFhem: heme and porphyrin; NRJatp: ATP-proton motive force

interconversion; NRJsug: sugars; OTHpha: phage related functions and prophages; PURpyr: pyrimidine ribonucleotide biosynthesis; PURsal: salvage of nucleosides and nucleotides; REGdeo: deoR-family regulators; REGgen: regulatory functions, general; TRDdeg: degradation of proteins, peptides and glycopeptides; TRDfac: translation factors; TRDsyn: ribosomal proteins synthesis and modification; TSPami: amino acids, peptides and amines; TSPani: anions;UNK: unknown.
