# Supplementary material for: A Genome-Scale Integration and Analysis of Lactococcus lactis Translation Data
Source: PLoS Comput Biol. 2013 Oct 10;9(10):e1003240. doi: 10.1371/journal.pcbi.1003240 (PMC3794899; doi:10.1371/journal.pcbi.1003240)
Supplement: Table S2 — Functional enrichment for genes with highest or lowest specific or absolute protein synthesis rate. (PDF) [file pcbi.1003240.s011.pdf]

Genes' group	Functional category	$n^{genes}_{per\ category}$
200 genes with highest V_{abs}	**TRD (p=1.2e-4**)	32/96
	ENV (p=6.0e-2)	12/42
	UNK (p=6.7e-2)	79/79
200 genes with lowest V_{abs}	**REG (p=2.8e-3)**	23/73
	**TSP (p=1.1e-2)**	28/103
	**NRJ (p=2.7e-2)**	25/96
	**AMI (p=4.5e-2)**	11/36
200 genes with highest V_{spec}	**OTH (p=4.9e-4)**	33/107
	**UNK (p=5.4e-4)**	90/384
	TRD (p=7.9e-2)	23/96
200 genes with lowest V_{spec}	**NRJ (p=1.7e-6)**	36/96
	**TSP (p=5.3e-3)**	29/103
	**REG (p=2.7e-2)**	20/73
	**ENV (p=2.7e.2)**	13/42
	AMI (p=9.7e-2)	10/36

Table S2: Functional enrichment for genes with highest or lowest specific or absolute protein synthesis rate.

AMI: amino acid biosynthesis, ENV: cell envelope, NRJ: energy metabolism, OTH: other categories, REG: regulatory functions, TRD: translation, TSP: transport and binding proteins, UNK: unknown function.
