# Supplementary material for: A Genome-Scale Integration and Analysis of Lactococcus lactis Translation Data
Source: PLoS Comput Biol. 2013 Oct 10;9(10):e1003240. doi: 10.1371/journal.pcbi.1003240 (PMC3794899; doi:10.1371/journal.pcbi.1003240)
Supplement: Text S1 — Supplementary methods. (PDF) [file pcbi.1003240.s012.pdf]

Supplementary Text S1: Supplementary methods for the paper "A Genome-Scale Integration and Analysis of *Lactococcus lactis* Translation Data"

Julien Racle[1,2], Flora Picard[3,4,5], Laurence Girbal[3,4,5], Muriel Cocaign-Bousquet[3,4,5,*] and Vassily Hatzimanikatis[1,2,*]
[1]Laboratory of Computational Systems Biotechnology, Ecole Polytechnique Fédérale de Lausanne (EPFL), CH-1015 Lausanne, Switzerland
[2]Swiss Institute of Bioinformatics (SIB), CH-1015 Lausanne, Switzerland
[3]Université de Toulouse; INSA, UPS, INP; LISBP, 135 Avenue de Rangueil, F-31077 Toulouse, France
[4]INRA, UMR792 Ingénierie des Systèmes Biologiques et des Procédés, F-31400 Toulouse, France
[5]CNRS, UMR5504, F-31400 Toulouse, France

*Corresponding authors: vassily.hatzimanikatis@epfl.ch(VH) and cocaign@insa-toulouse.fr(MCB)

Table of content

AVERAGE ELONGATION RATE CONSTANT 1
STOCHASTIC MODEL 2
DISTRIBUTION OF MRNA FRAGMENT SIZES 3
RECALIBRATING THE DATA 4
EXTENSION OF THE MODEL WITH AN INITIATION INHIBITING COMPLEX 6
SUPPL REFERENCES 6

Average elongation rate constant

Similarly to what has already been done for *E. coli* [1], we computed a cell-averaged translation elongation rate constant for *L. lactis* in the given condition. There for cells grown in exponential phase at a growth rate of 0.88 h^{-1}, it was measured that one gram of *L. lactis* dried cells contained 0.45 g of proteins and corresponded to 1.7 x 10^{12} cells, and the percentages of each amino acid in protein was also available [2]. This allows estimating the average mass of 1 amino acid, $\bar{m}_{aa} = 2.1 \cdot 10^{-22}\,\text{g}$, and also the mass of proteins present in a single cell: $m_{prot/cell} = 2.65 \cdot 10^{-13}\,\text{g} \cdot \text{cell}^{-1}$. With these two numbers we get the average number of proteins present in a cell: $\bar{N}_{aa/cell} = 1.26 \cdot 10^{9}\,\text{aa in proteins}$. Next the rate of incorporation of amino acids into proteins is given by:

$$\frac{d\bar{N}_{aa/cell}}{dt} = \frac{d(x \cdot M)}{dt} = \frac{x \cdot dM}{dt} = x \cdot \mu \cdot M = \mu \cdot \bar{N}_{aa/cell} \qquad \text{(S1)}$$

where M is the biomass and x represents the fraction of the biomass that is proteins (45 % of dry weight), and is considered as constant.

On the other hand, the proportion of active ribosomes was measured in *L. lactis* as $\beta_r = 0.61$ [3]. But no value for the total number of ribosomes was obtained, thus we assumed similar values to *E. coli* [1], interpolated to the growth rate from *L. lactis*; so $N_r = 21.7 \cdot 10^3$. Each of the active ribosome is then translating proteins with a mean insertion rate of amino acids, k_E (per ribosome). This gives another equation for the average incorporation rate of amino acids into all the proteins from the cell:

$$\frac{d\bar{N}_{aa/cell}}{dt} = \beta_r \cdot N_r \cdot k_E \tag{S2}$$

From equations S1 and S2, we obtain

$$k_E = \frac{\mu \cdot \bar{N}_{aa/cell}}{\beta_r \cdot N_r} = 23.3 \ \frac{\text{aa}}{\text{s} \cdot \text{ribosomes}} \tag{S3}$$

The value obtained here is similar to the values estimated for *E. coli*, ranging between 12 and 21 $\frac{\text{aa}}{s \cdot \text{ribosome}}$ for growth rates of 0.6 and 2,5 h^{-1} respectively. Note however that these values are only some average values, obtained when neglecting the protein decays, and the values could also change depending on the cell cycle.

Stochastic model

In the bigger part of this study, the model described by equations 1 – 6 (of the *main text*) is solved with help of deterministic equations, which correspond to the cells-average results. However, as we have seen (see *main text*), the experimentally observed distribution of polysome sizes for the genes is not expected and could not be modeled by such deterministic results. Therefore we inspected if the observed results could be better explained by stochastic simulations that will result in a distribution of the output values instead of a single average.

For this, we implemented a stochastic Monte Carlo algorithm that describes our model. Note that the equations 1 – 6 (from the *main text*) can easily by modified to a Monte Carlo scheme: the fluxes, V_i^l, will directly correspond to the reaction propensities,

describing the probability for a certain equation to happen, and the W_I^l, W_{i+1}^l terms in these equations need to be replaced by a check that the initiation region is free or that the next codon is free, respectively, in order to allow the ribosome binding or translocation. The results from these simulations for various values of initiation and termination rate constants are shown in Fig. S8. We observe that depending on the values of these rate constants, the distribution of polysome sizes can be more or less broad, but in no case is it possible to observe two peaks in these distributions, contrary to what is observed in the experimental data (Fig. S2).

Distribution of mRNA fragment sizes

To characterize the distribution of mRNA fragment sizes, we developed a simplified model of transcription and mRNA decay. In this model we considered a direct binding of the RNA polymerase to its promoter. Each gene was assumed to have a unique promoter site. The transcription elongation rate was assumed uniform. The model also allowed for the decay of full-sized mRNA chains. We therefore solved the following system of equations and determined the steady state values.

$$\begin{cases} \frac{dm_1^l}{dt} = k_{bind}^l \cdot RNAp_{free} \cdot W_I^l - k_{transcr} \cdot m_1^l \cdot W_{1+1}^l \\ \frac{dm_2^l}{dt} = k_{transcr} \cdot m_1^l \cdot W_{1+1}^l - k_{transcr} \cdot m_2^l \cdot W_{2+1}^l \\ \vdots \\ \frac{dm_{n^l}^l}{dt} = k_{transcr} \cdot m_{n^l-1}^l \cdot W_{n^l-1+1}^l - k_{term}^l \cdot m_{n^l}^l \\ \frac{dM_l}{dt} = k_{term}^l \cdot m_{n^l}^l - k_{decay}^l \cdot M_l \end{cases} \quad \text{(S4)}$$

where m_i^l is the number of fragments of mRNA species *l* of length *i* (these fragments are still bound to the RNA polymerase, *RNAp*); M_l is the number of full length mRNA of species *l*; $RNAp_{free}$ are the free RNA polymerases; W_I^l and W_{i+1}^l are the conditional probabilities of having the given DNA bp free, defined similarly than in equations 3 and 4 (from the *main text*); $k_{transcr}$ is the transcription rate constant and is given a similar value than translation elongation rate as diverse experiments observe concurrent transcription and translation having about the same rate (we use

$k_{transcr} = 20 \text{ codons/s} = 60 \text{ bp/s}$); k^{l}_{term} - the transcription termination constant - is taken equal to the transcription elongation to be non-rate limiting as has been observed; k^{l}_{decay} is the mRNA decay rate and is known for most of *L. lactis* genes from experiments [4]; and k^{l}_{bind} is the *RNAp* binding rate constant to the promoter of gene species *l*, the value of this rate was computed in order to have the steady state value of M_l matching the experimental measures of mRNA concentrations.

Recalibrating the data

As explained in the *main text*, the polysome size estimated directly from the experimental data for 129 genes showed values above the theoretically possible value (i.e. for example a gene with an ORF length of 110 codons, could in principle accommodate up to 11 ribosomes as one ribosome covers 10 codons, but some of the genes showed polysome size above their maximum). Therefore some refinement was needed in the data treatment. Using the distribution of the genes between the fractions (Fig. S2), it was observed that these *strange* genes had a high proportion of their mRNA in the last fraction, corresponding to a mean polysome size of 14. We hypothesized therefore that some copies of the mRNAs were being dragged along with the heavy complexes to the bottom of the sucrose gradient so that some copies of the mRNAs appeared to be more loaded than in reality. The last elution fraction was therefore assumed to collect some garbage along its true signal.

In order to gain more confidence in the results, this garbage was then redistributed among the various fractions with the following procedure (this was done for all genes, not only the ones that obviously had strange behaviors):

1. Denote G_i the proportion of mRNA copies from gene *i* that is present in the last elution fraction.
2. Compute:

$$P_{\max,i} = \min\left(\frac{n_i}{L}, P_{\max}^{allGenes}\right) \tag{S5}$$

   where $P_{max,i}$ represents the maximum possible polysome size for gene *i* as observed in the experiment, n_i is the ORF length of this gene, *L* is the number of

codons covered by a ribosome and $P_{\max}^{allGenes}$ is the maximal polysome size observed for the elution of all genes (observed as 17.9 ribosomes in this study [3]).

3. The mRNA in the garbage are assumed to have a uniform distribution between 0 and $P_{max,i}$ polysome, accounting the fact that the *garbage train* will drag everything with it, irrespectively of how many ribosomes are on the mRNA. Therefore, randomly draw N_{rand} values in the uniform interval $U\left(0,P_{\max,i}\right)$ and count how many of the values correspond to each elution fraction bounds. The proportion of the draws in fraction *j* for gene *i* is written: $f_{i,j}^{G}$.
4. Then we obtain the proportion of *signal mRNA*, $f_{i,j}^{s}$, in each fraction:

$$\begin{cases} f_{i,j}^{s} = f_{i,j} + f_{i,j}^{G} & j = [B-G] \\ f_{i,j}^{s} = f_{i,j}^{G} & j = H \end{cases} \tag{S6}$$

where $f_{i,j}$ is the proportion of mRNA copies from species *i* observed in fraction *j* in the experiment.

5. Then, similarly as was done in [3], we perform some bootstrap analysis to assign a peak fraction for each gene, but using the new distribution of the gene's mRNA, $f_{i,j}^{s}$.

This procedure allows gaining confidence in the results (no more gene is observed at a polysome size bigger than possible) and it does not lead to any bias: it does not artificially remove any possible ribosomal density.

Recalibration for the stressed L. lactis *condition*

This recalibration procedure can be applied for the data coming from all experimental conditions. However, note that for our data in the stress condition, only 5 elution fractions were collected instead of the previous 7, in order to have sufficient amount of mRNA in each fraction. Thereby, the last 2 fractions were merged together. And as we observed in the normal condition that it was sufficient to consider that garbage was collected in the very last fraction, we assumed the same was true here and therefore assumed only 50% of the mRNA present in the last "double-fraction" was coming from garbage. This was sufficient in order to remove all the genes that had a too high polysome size with respect to their length.

Extension of the model with an initiation inhibiting complex

As explained in the *main text*, the experimental observations made us hypothesize that some complex might be inhibiting the translation initiation, once this complex is bound near of the initiation site. We then tested this with a simplified extension of our stochastic simulations (Fig. S9).

For this, we assumed that once a ribosome has initiated, it can continue its translation elongation in the exact same way as in the previous model (see *main text* and *above*). On the other hand, we added a step that was competing with the translation initiation, where instead of the ribosome binding to the initiation site, we made it possible for another complex to bind the initiation region (Fig. S9A). Once such a complex was bound, we assumed that no further translation initiation could happen, as long as this other complex was bound. For simplicity, we also assumed that when a ribosome was still in the initiation region (the first L codons of the mRNA), it was blocking the binding of this other complex, in the same way that this ribosome is blocking the binding of a second ribosome.

The results obtained with this inhibiting complex for various values of the parameters are in good qualitative agreement with experimental observations (Fig. S9B-D and Fig. S2).

Suppl REFERENCES

1. Bremer H, Dennis PP (1996) Modulation of chemical composition and other parameters of the cell by growth rate. In Escherichia coli and Salmonella typhimurium: Cellular and Molecular Biology. Edited by Neidhardt JC, Low KB, Magasanik B et al Washington DC: ASM;: 1527-1542.
2. Novak L, Loubiere P (2000) The metabolic network of Lactococcus lactis: distribution of (14)C-labeled substrates between catabolic and anabolic pathways. J Bacteriol 182: 1136-1143.
3. Picard F, Milhem H, Loubière P, Laurent B, Cocaign-Bousquet M, et al. (2012) Bacterial translational regulations: high diversity between all mRNAs and major role in gene expression. BMC Genomics 13: 528.
4. Redon E, Loubiere P, Cocaign-Bousquet M (2005) Role of mRNA stability during genome-wide adaptation of Lactococcus lactis to carbon starvation. J Biol Chem 280: 36380-36385.
